# Ionic currents influencing spontaneous firing and pacemaker frequency in dopamine neurons of the ventrolateral periaqueductal gray and dorsal raphe nucleus (vlPAG/DRN): A voltage-clamp and computational modelling study

**DOI:** 10.1007/s10827-017-0641-0

**Published:** 2017-04-03

**Authors:** Antonios G. Dougalis, Gillian A. C. Matthews, Birgit Liss, Mark A. Ungless

**Affiliations:** 1 MRC London Institute of Medical Sciences (LMS), Du Cane Road, London, W12 0NN UK; 2 Institute of Clinical Sciences (ICS), Imperial College London, Faculty of Medicine, Du Cane Road, London, W12 0NN UK; 3 Institute of Applied Physiology, University of Ulm, Faculty of Medicine, 89073 Ulm, Germany; 4 Picower Institute for Learning and Memory, Department of Brain and Cognitive Sciences, Massachusetts Institute of Technology, Cambridge, MA 02139 USA

**Keywords:** Autorhythmicity, Electrophysiology, Delayed rectifier, Persistent sodium current, Depolarization block

## Abstract

Dopamine (DA) neurons of the ventrolateral periaqueductal gray (vlPAG) and dorsal raphe nucleus (DRN) fire spontaneous action potentials (APs) at slow, regular patterns *in vitro* but a detailed account of their intrinsic membrane properties responsible for spontaneous firing is currently lacking. To resolve this, we performed a voltage-clamp electrophysiological study in brain slices to describe their major ionic currents and then constructed a computer model and used simulations to understand the mechanisms behind autorhythmicity *in silico*. We found that vlPAG/DRN DA neurons exhibit a number of voltage-dependent currents activating in the subthreshold range including, a hyperpolarization-activated cation current (I_H_), a transient, A-type, potassium current (I_A_), a background, ‘persistent’ (I_NaP_) sodium current and a transient, low voltage activated (LVA) calcium current (I_CaLVA_). Brain slice pharmacology, in good agreement with computer simulations, showed that spontaneous firing occurred independently of I_H_, I_A_ or calcium currents. In contrast, when blocking sodium currents, spontaneous firing ceased and a stable, non-oscillating membrane potential below AP threshold was attained. Using the DA neuron model we further show that calcium currents exhibit little activation (compared to sodium) during the interspike interval (ISI) repolarization while, any individual potassium current alone, whose blockade positively modulated AP firing frequency, is not required for spontaneous firing. Instead, blockade of a number of potassium currents simultaneously is necessary to eliminate autorhythmicity. Repolarization during ISI is mediated initially *via* the deactivation of the delayed rectifier potassium current, while a sodium background ‘persistent’ current is essentially indispensable for autorhythmicity by driving repolarization towards AP threshold.

**Electronic supplementary material:**

The online version of this article (doi:10.1007/s10827-017-0641-0) contains supplementary material, which is available to authorized users.

## Introduction

Studies of physiological characteristics of dopamine (DA) neurons have been largely concentrated around the midbrain areas of substantia nigra pars compacta (SNc) and ventral tegmental area (VTA) due to their well-known importance in motor control and reward processing (Schultz 1998; Pollack 2001). Midbrain DA neurons fire spontaneous action potentials (APs) at 3–8 Hz in a tonic or bursting manner *in vivo* (Grace and Bunney 1983a, 1983b; Grace and Bunney 1984a, 1984b) and maintain spontaneous tonic (but not bursting) firing at 1–10 Hz even in the absence of synaptic transmission *in vitro* (Grace and Onn 1989; Lacey et al. 1989; Puopolo et al. 2007; Lammel et al. 2008; Khaliq and Bean 2010). Through intensive research using the *in vitro* brain slice preparation and/or dissociated cell preparations a number of intrinsic ionic currents have been described to modulate firing frequency of midbrain DA neurons including, a hyperpolarization-activated cation current [I_H_] (Seutin et al. 2001; Neuhoff et al. 2002), an A-type potassium current [I_A_] (Liss et al. 2001; Koyama and Appel 2006a; Khaliq and Bean 2008; Kimm and Bean 2014; Subramaniam et al. 2014), an M-type potassium current [I_M_] (Hansen et al. 2006; Koyama and Appel 2006b; Koyama et al. 2007; Drion et al. 2010), an apamin-sensitive, calcium-activated potassium current [I_SK_] (Wolfart et al. 2001; Koyama et al. 2005; Bishop et al. 2010; Deignan et al. 2012), an iberiotoxin-sensitive calcium-activated potassium current [I_BK_] (Kimm et al. 2015), a delayed rectifier potassium current [I_Kdr_] (Silva et al. 1990; Kimm et al. 2015), transient (low voltage activated, LVA) and persistent (high voltage activated, HVA) calcium currents [I_CaLVA_ and I_CaHVA_] (Kang and Kitai 1993a, 1993b; Wolfart and Roeper 2002; Branch et al. 2014; Poetschke et al. 2015; Philippart et al. 2016) and background ‘persistent’ and transient sodium currents [I_NaP_ and I_NaT_] (Puopolo et al. 2007; Khaliq and Bean 2010; Ding et al. 2011).

The tonic-firing pacemaking mechanism of SNc and VTA midbrain DA neurons seem to differ in at least two important ways. First, I_H_ current inhibition decreases pacemaker firing frequency in some SNc neurons but not in VTA neurons (Neuhoff et al. 2002; Khaliq and Bean 2010) arguing that I_H_ current may have a role in pacemaking in SNc neurons but it is not essential for autorhythmicity in either group. It is noteworthy that VTA neurons, especially those projecting to the prefrontal cortex, lack a prominent expression of an I_H_ current (Margolis et al. 2006; Lammel et al. 2008). Second, during the interspike interval (ISI), SNc neurons rely substantially on subthreshold calcium conductances to repolarise to AP threshold (Wilson and Callaway 2000; Chan et al. 2007; Puopolo et al. 2007; Putzier et al. 2009) and to a far lesser degree on sodium conductances (Puopolo et al. 2007; Khaliq and Bean 2010). Interestingly, the opposite is true for VTA neurons that, rely heavily on both voltage-dependent, TTX-sensitive and voltage-independent, TTX-resistant, background ‘persistent’ sodium currents to repolarise to AP threshold during ISI (Khaliq and Bean 2010). SNc pacemaker firing frequency can be often greatly reduced or even halted by L-type calcium channel blockers (Nedergaard et al. 1993; Mercuri et al. 1994; Puopolo et al. 2007; Putzier et al. 2009, but see also Chan et al. 2007, Guzman et al. 2009, Poetschke et al. 2015) or abolished by cadmium (Puopolo et al. 2007) suggesting that calcium currents are indispensable for SNc autorhythmicity although the impact of L-type blockers on firing rate of SNc DA neurons is often hard to reproduce for reasons that are not clear (see Chan et al. 2007; Guzman et al. 2009; see also explanations provided in modeling study by Drion et al. 2011). In contrast, in VTA neurons, the absence of external calcium speeds rather than abolishes the pacemaker firing frequency (Khaliq and Bean 2010), whereas blockade of a TTX-sensitive sodium current resulted in a stable resting membrane potential (negative to AP threshold) with no evidence of background oscillations, a situation unlike to what has been reported for SNc neurons that exhibit calcium-mediated small oscillating potentials (SOP) in the presence of sodium channel blockers (Nedergaard et al. 1993; Mercuri et al. 1994; Chan et al. 2007; Puopolo et al. 2007; Guzman et al. 2009). The presumed qualitative difference in the mechanism of pacemaking in VTA neurons and SNc neurons, in that there is little role of subthreshold calcium current in driving pacemaking in VTA neurons, also supports the hypothesis that the selective vulnerability of SNc DA neurons in Parkinson’s disease (as opposed to the selective sparring of VTA neurons, see Mosharov et al. 2009; Surmeier and Schumacker 2013) may depend on this prominent calcium entry during pacemaking cycles and its link to mitochondrial potential flickering and oxidative stress (Guzman et al. 2010; Surmeier et al. 2011; Philippart et al. 2016). Given the diversity of the functional phenotypes of midbrain DA neurons based on their neuroanatomical positioning and projection targets (Lammel et al. 2008, 2011; Poulin et al. 2014; Beier et al. 2015; Lerner et al. 2015), it is likely that the VTA and SNc DA neuronal subgroups may utilise a multitude of mechanisms under different circumstances to maintain tonic firing (*e.g.* see results in Chan et al. 2007; Puopolo et al. 2007; Guzman et al. 2009; Poetschke et al. 2015) which in turn could influence to a different degree the neuron’s propensity for degeneration.

The ventrolateral periaqueductal grey (vlPAG) and dorsal raphe nucleus (DRN) regions contain a population of DA neurons, often considered to be a dorsocaudal extension of the VTA area towards the brainstem, termed collectively the A10dc system (Hokfelt et al. 1984). These DA neurons may play a role in wakefulness (Lu et al. 2006), opiate antinociception (Flores et al. 2004; Li et al. 2016), drug reward (Flores et al. 2006; Li et al. 2013) while they have been recently shown to encode the experience of social isolation (Matthews et al. 2016). Using the pitx3-GFP and TH-GFP transgenic mouse models as an aid to identification of DA neuron phenotype, we have previously reported the physiological characteristics of DA vlPAG/DRN neurons *in vitro* under current-clamp (regular firing with broad APs at a range 1-10 Hz and spike-frequency adaptation in response to prolonged depolarization, Dougalis et al. 2012). Interestingly during our studies we have also noted that spontaneous tonic firing of DA vlPAG/DRN neurons *in vitro* can persist in the absence of glutamatergic and GABAergic synaptic transmission but the mechanism behind this property is not known despite the availability of such data for SNc and VTA, midbrain DA neuronal groups (see Grace and Onn 1989; Silva et al. 1990; Puopolo et al. 2007; Khaliq and Bean 2010). To resolve this, we have conducted a voltage-clamp study to provide a kinetic description of major sodium, potassium and calcium ionic currents operant on DA vlPAG/DRN neurons in brain slices. Based on experimentally derived voltage-clamp data, we then constructed a simplified, conductance-based, Hodgkin and Huxley-type, computer model and validated its behaviour against *in vitro* neurophysiological data. Using simulations in the computational DA model, we explored the contribution of individual ionic currents in vlPAG/DRN DA neuron’s spontaneous firing, pacemaker frequency and threshold for spike frequency adaptation *in silico*.

## Methods

### Brain slice preparation

Three to six months old, male, GFP-pitx3 heterozygous mice (transgenic line generated as described previously, see Zhao et al. 2004) were sacrificed by isoflurane anaesthesia followed by decapitation. All breeding and experimental procedures were conducted in accordance with the Animals (Scientific Procedures) Act of 1986 (United Kingdom) or were approved by the German Regierungspräsidium Tübingen (AZ 35/9185.81–3. TV1043, Reg. Nr. 0.147). The brain was rapidly removed out of the cranial cavity and bathed with ice-cold (0–2 °C), fully equilibrated (with carbogen gas, 95% carbon dioxide and 5% oxygen) artificial celebrospinal fluid (aCSF, composition in mM, NaCl 120, KCl 3.5, NaH_2_PO_4_ 1.25, NaHCO_3_ 26, Glucose 10, MgCl_2_ 1, CaCl_2_ 2). Two or three thin coronal brain slices (220 μm thickness) encompassing the dorsal raphe nucleus (DRN) were obtained using a vibratome (Leica VT1000S, Germany) and were maintained into a standard in-house-made maintenance chamber (Edwards et al. 1989) gently and continuously aerated with carbogen gas for at least one and a half hours at room temperature (20–22 °C) before use for electrophysiology.

### Neuron identification

Slices were transferred to a submersion recording chamber and were continuously perfused at a rate of 2–4 ml/min with fully oxygenated aCSF (composition as above) at 35.5 °C (± 0.5 °C) for at least 30 min before attempting recordings. Whole-cell current and voltage-clamp recordings were obtained from DA neurons using infra-red differential interference contract (IR-DIC) videomicroscopy as described previously (Stuart et al. 1993). In short, neurons were visualized under an upright microscope equipped with ×40 or ×60 objectives, an IR filter, DIC optics and a charged cooled diode (CCD) video camera. GFP-pitx3 DA neurons were firstly identified in the slice by using fluorescence illumination coupled to a GFP excitation filter and then further visualized under IR-DIC conditions prior to electrophysiology. For comparative purposes, in some experiments, we also obtained targeted recordings from SNc and VTA DA neurons as described previously (Bishop et al. 2010).

### Electrophysiology

Tight seal (>10 GΩ) whole-cell, current-clamp recordings were performed with a Multiclamp 700B amplifier (Molecular Devices), while voltage-clamp recordings were performed with an Axopatch 200A (Molecular Devices) or an EPC-10 (HEKA Electronics, Germany) using glass microelectrodes (3–6 MΩ in resistance) filled with a standard internal solution containing (in mM) 140 potassium gluconate (KGlu), 5 NaCl, 1 MgCl_2_, 10 HEPES, 1 EGTA, 2 MgATP and 0.5 LiGTP, pH 7.25–7.35, osmolality 280–290 mosmol/l. For voltage-clamp recordings aiming to isolate specific ionic currents, internal and external solutions were modified accordingly depending on the current under investigation (see descriptive text below in solutions and voltage-clamp protocols for ionic currents). Series resistance (R_s_) and input resistance (R_in_) were frequently monitored throughout the experiments *via* at 10 mV, 250 ms hyperpolarizing step under voltage-clamp. R_s_ value was <13 MΩ (average 8.3 ± 1.1 MΩ, *n* = 40) for all recordings and was compensated by approximately 60–90% leaving on average an uncompensated Rs of 3.2 MΩ (range, 2.5–7 MΩ) and an estimated error in voltage-clamp command potentials when recording an one nanoampere current under most circumstances of <5 mV. Whole-cell capacitive transients were evaluated under voltage-clamp through a 10 mV step (−50 to −60 mV) and were negated using the amplifier’s circuitry. The recorded neurons had an average C_W_ of 7.8 ± 0.4 pF (*n* = 40, range, 4.5 to 12.0 pF). Currents were filtered at 1 KHz (low-pass), collected at 3–5 KHz through an A/D converter (PC-5230, National Instruments, USA) on WINWCP (University of Strathclyde, courtesy of Dr. John Dempster) or *via* an onboard card from EPC-10 to PatchMaster software (HEKA Electronics, Germany) and stored for offline analysis. The combination of our intracellular and extracellular recording solutions lead to an estimated liquid junction potential (LJP) of +7 and +12 mV for the cesium methanesulphonate (CsMe) and KGlu filled electrodes respectively (measured in pClamp10 calculator). Holding voltages reported herein for electrophysiological experiments have been corrected by this amount.

### Solutions and pharmacology

The standard KGlu internal solution (detailed above) was used in voltage-clamp experiments isolating sustained (I_Kdr_ and I_M_) and transient (I_A_) potassium currents as well as the hyperpolarization-activated cation current (I_H_). In these experiments the aCSF was supplemented with tetrodotoxin (TTX, 1 μM). For experiments involving calcium, barium and sodium current isolation, KGlu was substituted for CsMe (120 mM) and tetraethylammonium (TEA, 20 mM) in internal solutions. External aCSF solution’s NaCl concentration was reduced to 100 mM and was supplemented with 20 mM TEA, 2 mM 4-aminopyridine (4-AP) and 1 μM TTX for calcium/barium current recordings. Barium chloride (BaCl_2_) substituted calcium chloride in the external solution for barium current recordings (1 mM BaCl_2_ for 2 mM CaCl_2_). For isolation of sodium currents we included 1 mM BaCl_2_ in external aCSF and replaced TTX with cadmium chloride (200 μM). Osmolality in all above aCSF solutions was kept constant at 300 mosmol/l by equivalent reductions in NaCl when more than 2 mM additions were made. All drugs used in this study for formulating external and internal solutions were bought from Sigma-Aldrich (UK) apart from 2,3-dihydroxy-6-nitro-7-sulfamoyl-benzo[f]quinoxaline-2,3-dione (NBQX), tetrodotoxin (TTX), 10,10-*bis*(4-Pyridinylmethyl)-9(10*H*)-anthracenone dihydrochloride (XE991) and 4-Ethylphenylamino-1,2-dimethyl-6-ethylaminopyrimidinine (ZD7288) which were bought from Tocris (UK). For pharmacological investigations, drugs were made fresh from appropriately concentrated aliquots stored according to the manufacturer’s recommendations. All drugs were dissolved on the day in freshly prepared oxygenated aCSF and superfused on the tissue for 10 min to achieve steady-state concentration in the intima of the brain slices before any measurements were taken.

### Voltage-clamp protocols for ionic currents

#### Hyperpolarization-activated cation current (I_H_)

Neurons were voltage-clamped at −47 mV and hyperpolarizing steps of 1 s in duration were delivered in 10 mV decrements from −62 to −152 mV in the presence of TTX (1 μM) using a standard KGlu based-internal solution. Determination of I_H_ current amplitude at potentials more negative than −152 mV was not routinely attempted as this lead to loss of recording. I_H_ currents were measured as slowly activating inward currents calculated from the difference of the instantaneous currents and the steady-state currents, in the first 5–20 ms after the beginning and 25 ms before the end of the hyperpolarizing voltage step respectively, at each test holding potential.

#### A-type (I_A_) and delayed rectifier (I_Kdr_) potassium currents

I_A_ currents were isolated by digital subtraction of currents between two specific voltage-clamp protocols using a standard KGlu based-internal solution in the presence of TTX (1 μM) as reported previously (Koyama and Appel 2006a). I_A_ current amplitude was measured from peak till 25 ms before the end of the inactivating current during the 1 s long pulses used in our study. To study I_A_ current steady-state activation, neurons were held at −72 mV and a 250 ms hyperpolarizing prepulse to −112 mV was used to facilitate the recovery from inactivation of the I_A_ current. Subsequently the neurons were stepped to the test voltage from −92 to +18 mV in 10 mV increments for 1 s to activate and record the resultant I_A_ current transient and its inactivation (protocol 1). In the second protocol, neurons were held at −72 mV and a 250 ms prepulse to −52 mV was given to facilitate the inactivation of the I_A_ transient current before stepping the neurons from −92 to +18 mV in 10 mV increments for 1 s (protocol 2). Currents obtained from the second protocol were slowly activating sustained outward currents reminiscent of a delayed rectifier (I_Kdr_) and were subtracted from the currents obtained from protocol 1 to obtain the I_A_ current. To study I_A_ current steady-state inactivation, neurons were held at −47 mV and a 250 ms prepulse was given from -152 mV to −52 mV (in 10 mV increments) before stepping the neuron to the test voltage of −47 mV for 1 s to record the extend of I_A_ current inactivation as a function of the prepulse holding voltage. Current subtraction was not necessary for steady-state inactivation experiments because the fast peak of I_A_ current was not contaminated by the slowly activating sustained currents at the test voltage of −47 mV. We also obtained I_A_ currents *via* a single activation voltage protocol (protocol 1 described above) in the presence of 10 mM TEA (to block I_Kdr_ potassium currents) as described previously (Silva et al. 1990) to compare the results obtained *via* the two protocol subtraction method. Finally, I_A_ currents were also isolated *via* a third method using protocol 1 above in the presence of TEA (10 mM) by current subtraction after the addition of 2 mM 4-AP to reveal the 4-AP sensitive component. I_Kdr_ currents were isolated using a KGlu based-internal solution in the presence of TTX (1 μM) as reported previously (Silva et al. 1990) using a modified protocol 2 detailed above. To study I_Kdr_ current steady-state activation neurons were held at −72 mV and a 250 ms hyperpolarizing prepulse to −52 mV was used to facilitate the inactivation of the I_A_ transient current. Subsequently the neurons were stepped to the test voltage from −92 to +18 mV in 10 mV increments for 1 s to activate and record the resultant slowly-activating sustained I_Kdr_ current. Current amplitude was measured 25 ms before the end of the depolarizing step.

#### M-type (I_M_) potassium currents

I_M_currents were isolated using a standard deactivation protocol in KGlu-based internal solution in the presence of TTX (1 μM) as reported previously (Koyama and Appel 2006b). The neurons were held at −72 mV and were given a depolarizing prepulse to −32 mV for 1 s before stepped down to the test voltage from −42 to −72 mV in 10 mV decrements for 1 s to record the resultant I_M_ current deactivation tail.

#### Calcium and Barium currents (I_Ca_ and I_Ba_)

Low and high threshold voltage-activated (LVA and HVA) inward calcium/barium currents (LVA and HVA) were isolated using a combination of blockers in both internal and external solutions in an attempt to block potassium and sodium conductances as described previously (Brevi et al. 2001). Neurons were bathed in TTX (1 μM), 4-AP (2 mM) and TEA (20 mM) and recorded with CsMe (120 mM) and TEA (20 mM) based internal solutions. For I_Ca_ experiments, neurons were held at −67 mV and were depolarized to the test voltage from −57 to +3 mV in 10 mV increments for 250 ms. Calcium currents recorded through this protocol would inactivate with different time constants during our steps at nearly all potentials. This inactivation could theoretically result from calcium-mediated intracellular calcium release (that can induce calcium channel inactivation) or it could be directly related to the kinetic, voltage-dependent properties of the calcium channels (Giannattasio et al. 1991; Haack and Rosenberg 1994; Catterall 2000; Budde et al. 2002). To reduce the amount of inactivation seen with calcium and discriminate better between these two possibilities we have used barium ions (1 mM) instead of calcium (2 mM) as the charge carrier, since barium currents undergo significantly less inactivation during long steps than calcium currents (see Hille 2001). Furthermore, to ascertain the presence of LVA currents we recorded barium currents from a more hyperpolarized potential (−87 mV). I_Ba_ were recorded *via* two different protocols essentially used to isolate LVA/HVA or HVA currents alone (Brevi et al. 2001). Neurons were held at either −87 mV (LVA/HVA protocol) or −67 mV (HVA protocol) and were depolarized in 10 mV increments for 250 ms to +3 mV. Current subtraction was used to isolate the LVA component from LVA/HVA protocol by subtracting the HVA protocol currents. As an alternative method to study calcium and barium currents, we employed fast voltage-ramps (200–500 mV/s, from −107 to +13 mV) to measure the background flowing currents at different potentials after leak current subtraction.

#### Sodium currents (I_Na_)

Fast transient (I_NaT_) and persistent (I_NaP_) sodium currents were recorded from neurons bathed in TEA (20 mM), barium chloride (1 mM) and cadmium chloride (200 μM) using CsMe/TEA (120/20 mM)-based internal solutions as reported previously (Pignatelli et al. 2005; Magistretti et al. 2006). Neurons were held at −107 mV and a depolarizing prepulse of 5 ms in duration was delivered to −47 mV to activate unclamped axonal sodium currents, before hyperpolarizing the neuron to −77 mV for 4 ms and then step wise depolarizing it (in 5 mV increments from −77 to +13 mV for 110 ms) to sample somatic I_Na_ current activation devoid of axonal contamination as described previously (Milescu et al. 2010). To study steady-state inactivation of transient somatic sodium currents, neurons were subjected to the above protocol with a prepulse to −47 to activate and inactivate axonal sodium currents but then were given a 4 ms variable hyperpolarizing step from −77 to −42 mV (in 5 mV steps) before measuring the somatic sodium current at the test potential of −37 mV (held for 20 ms). The amplitude of the background ‘persistent’ sodium current was measured 10 ms before the end of the depolarizing steps and of the transient sodium current at the peak of the response during the steady state activation/inactivation protocol. We also sampled persistent non-inactivating sodium currents *via* an alternative method by subjecting neurons to slow voltage ramps (16 mV/s, from −107 to +53 mV) and leak current substraction in the presence of the above blocking solutions.

### Current-clamp experiments

The standard KGlu internal solution and standard external aCSF (as detailed above) were used in all current-clamp experiments unless otherwise indicated in text. Experiments were conducted in the presence of NBQX (5 μM) and picrotoxin (25 μM) to block excitatory and inhibitory synaptic activity respectively.

### Voltage-clamp data analysis

Voltage-clamped currents were averaged in three to five trials at each test holding voltage. We employed capacitive transient artifact and leak subtraction for sodium and calcium inward conductance using a modified P/4 protocol off-line. Averaged currents from different protocols were exported as text files (.txt) for subtraction in Excel spreadsheets and were then imported into Spike2 (Cambridge electronic design, CED, UK) for measurements and exponential function fitting. Data transformations, statistical analysis and fitting of single order Boltzmann function to the data set were performed in Prism software (version 7, GraphPad, USA).

Measured current amplitude at peak (or steady-state) was converted into conductance at any given holding potential by using eq. (1) below

1 $$ G=\frac{I}{V- Erev} $$

where, G represents the current conductance at a given test holding voltage V, while I is the current amplitude at the given holding voltage and E_rev_ is the equilibrium potential (calculated using the Nernst equation) of the current under investigation. The Nernst equation yielded for our conditions (35 °C) an estimated equilibrium potential of −105, +135, +75 mV for the major ions (E_K+_, E_Ca++_, E_Na+_, respectively).

To construct steady-state activation (or inactivation) curves we normalized conductance to its maximal value and plotted it against holding voltage (G/G_max_ against holding voltage). The data were then fitted with a single first order Boltzmann function using eq. (2) below

2 $$ G=\frac{G \max + G \min }{1+ \exp \left(\frac{V50- V}{s}\right)}+ G \min $$

where, G is the conductance at the holding test voltage of V, G_max_ is the maximal conductance set to be 1 and G_min_ is the minimum conductance set to be 0, V_50_ is the membrane potential for half-maximal activation and s is the slope factor of the activation/inactivation curve.

To measure the activation and inactivation time constants currents were fitted from start to peak (activation time constant) or from peak to end (inactivation time constant) with a single exponential function according to the eq. (3) below

3 $$ I=\operatorname{Im}{\mathrm{ax}}^{\ast } \exp \left(\raisebox{1ex}{$- t$}\!\left/ \!\raisebox{-1ex}{$\tau $}\right.\right) $$

where, I is the current amplitude at time t and I_max_ is the maximal current amplitude, while τ is the time constant. In some currents (*e.g.* sodium currents and A-type potassium currents), inactivation was best described by two exponential function fitting at most holding voltages. These currents were fitted accordingly with following eq. (4) for bi-exponential inactivation

4 $$ I=\operatorname{Im}\mathrm{ax}{\left(\mathrm{f}\right)}^{\ast } \exp \left(\raisebox{1ex}{$- t$}\!\left/ \!\raisebox{-1ex}{$\tau f$}\right.\right)+\operatorname{Im}\mathrm{ax}{\left(\mathrm{s}\right)}^{\ast } \exp \left(\raisebox{1ex}{$- t$}\!\left/ \!\raisebox{-1ex}{$\tau s$}\right.\right) $$

where, I is the total current amplitude at time t, I_max(f)_ is the maximal current amplitude of the first (fast) inactivating component of the current and I_max(s)_ is the maximal current amplitude of the second (slow) inactivating component of the current, while τ_f_ and τ_s_ are the fast and the slow component inactivation time constants respectively. Decision on using mono *versus* bi-exponential fitting was guided by inspection of the residual value table and compared by using the R value and the standard deviation for the fit. Calculated time constants were plotted against holding voltage and were fitted a single order modified first order Boltzmann function [eq. (5)] to calculate the voltage to attain half-maximal activation/inactivation time constant (V_50_ value) and its corresponding slope factor.

5 $$ \tau =\frac{\tau \max -\tau \min }{1+ \exp \left(\frac{V50- V}{s}\right)}+\tau \min $$

where, τ is the activation/inactivation time constant at the holding test voltage of V, τ_max_ is the maximal value of the time constant, τ_min_ is the minimum value of the time constant, V_50_ is the membrane potential where time constant is half maximal and s is the slope factor of the time constant curve.

### Current-clamp data analysis

All neurons were monitored frequently for stability of series resistance (R_s_) and input resistance through the recording and during pharmacological investigations. R_s_ did not exceed 25 MΩ in our recordings. Action potentials (APs) and accompanying afterhyperpolarization (AHP) were averaged during epochs of interest (100–200 APs) in Spike2 software (CED, UK) and their characteristics were calculated (AP amplitude, AP width, AHP amplitude, AHP repolarization) with respect to the AP threshold (defined as the point where the rate of voltage change exceeded 10 mV/ms, calculated through the first differential of the voltage trace). Average firing rate for each cell was calculated from the inverse of the average interspike interval (ISI) from ISI histograms while the coefficient of variation of the ISI was calculated as the ratio of the standard deviation (SD) of the ISI to the mean ISI. Hyperpolarizing current injections (−10 to −120 pA, 1000 ms in duration) were used to ascertain the extent of I_H_ current activation (referred to as the I_H_ current-mediated voltage-sag) and of the extent of I_A_ current activation (referred to as delayed repolarization). The voltage-sag was measured during the hyperpolarizing step as the voltage difference from the peak of the voltage response at the beginning of the step (occurring in the first 50–100 ms from step initiation) till 20 ms before the end of the hyperpolarizing step. Delayed repolarization was measured at the termination of the hyperpolarizing step as the time from step termination till the firing of the first AP. Depolarizing current injections (+10 to +120 pA, 1000 ms in duration) were used to elicit APs and measure the gain (input-output relationship) of vlPAG/DRN DA neurons. We measured instantaneous firing frequency by using the first two APs elicited by the depolarizing step. Sustained firing frequency was measured as the mean AP firing frequency during the last 100 ms of the depolarizing step as described previously (Dougalis et al. 2012).

### Statistical data analysis

All values reported represent mean ± standard error of the mean (s.e.m). The n number reported for each experiment represents replicated observations in different neurons from slices obtained from a minimum of three different animals (range of 3–5 per experiment as indicated in results section for each experiment). A *P* value of less than 0.05 was taken to indicate significance using a paired or unpaired t-test as appropriate for pair-wise comparisons or one-way analysis of variance (ANOVA) followed by Tukey’s multiple comparison test.

### Modelling and computer simulations

The DA neuron was modelled using NEURON software (Hines and Carnevale 1997, available freely at https://www.neuron.yale.edu/neuron/) as a single compartment, spherical neuron of 15 μm in diameter. General model assumptions and specific model equations used in the construction of the model are detailed in the appendix. The model code will be deposited and will be freely available through the modelDB database (https://senselab.med.yale.edu/modeldb/) following article publication (also available through direct request to the authors). Electrophysiological data were collected at 35 °C and thus the model replicates behaviour at this temperature. The model DA neuron was constructed to operate through eight Hodgkin and Huxley-type conductances (I_NaT_, I_NaP_, I_Kdr_, I_A_, I_M_, I_H_, I_CaHVA_, I_CaLVA_) and a leak conductance. Since it is not possible to examine electrophysiologically a single neuron for all conductances, the values derived for any conductance represent mean values over a population of different neurons examined and these were used in the model unless otherwise stated. All values used in the model (Table 1) were mean values taken from experimental data. In some cases, values were optimized by allowing use of mean ± one standard deviation of the reported experimental values where necessary to improve model functionality. For the operation of the model and in line with Hodgkin and Huxley (1952) we have used three activation gates for sodium currents and four activation gates for the delayed rectifier potassium current. Only one activation gate was used for calcium currents, I_A_ current, I_M_ and I_H_ current as described previously (Xiao et al. 2004) and in line with our experimental fitting. Barium currents were shifted 10 mV more positive to mirror the V_50_ values for calcium currents. The leak current in the DA model was given a constant conductance of 0.04 mS/cm^2^ and an E_rev_ of −55 mV based on experimental data of DA neurons mean input resistance (7 GΩ) and reversal potential in the presence of calcium, sodium and potassium channel blockers. Simulated responses (numerical integration in steps of 25 μs corresponding to a reconstruction frequency of 40 KHz) for the model DA neuron were exported programmatically from NEURON as text files (.txt) and imported in Spike2 (CED, UK) for measurements of membrane potential, firing frequency and action potential characteristics under basal and various test conditions.

**Table 1:** A summary of parameters utilized by the vlPAG/DRN DA model neuron

Current	E_rev_ (mV)	G_max_ (mS/cm^2^)	Activation function (A)		Inactivation function (B)		Activation function time constant (τ_A_)		Inactivation function time constant (τ_B_)	
			V_50_ (mV)	slope	V_50_ (mV)	slope	V_50_ (mV)	slope	V_50_ (mV)	Slope
I_A_	-73	6	-57.5	7.7	-93	-6.1	-68.8	-5.0	-24.6	-8.6
I_Kdr_	-73	3	-25.0	12.0	NA	NA	-38.4	-6.9	NA	NA
I_M_	-73	1	-35.0	8.5	NA	NA	-27.9	-6.9	NA	NA
I_NaT_	+50	20	-44.0	4.5	-62.0	-6.5	-28.0	-7.0	-14.5	-9.5
I_NaP_	+50	0.02	-57.0	3.5	NA	NA	#	#	NA	NA
I_CaHVA_	+120	0.04	-22.0	5.0	-40.0	-7.0	-40.0	-3	-39.0	-2.6
I_CLVA_	+120	0.04	-57.5	6.5	-83.0*	-6.1*	-68.8	-5.0	-24.6*	-8.6*
I_H_	-40	0.08	-114.7	-12.8	NA	NA	-112.7	6.7	NA	NA
I_leak_	-55	0.04	NA							

The values of reversal potential (E_rev_) and maximal conductance (G_max_) for each ionic current in the model DA neuron are presented (columns two and three). Columns four to seven detail the mean half-maximal (V_50_) and slope values for the voltage-dependence of the extent of activation/inactivation of the gating functions and voltage-dependence of the rate of activation/inactivation of the gating functions derived by first order Boltzmann function fitting to the electrophysiological data (see methods). All values have been corrected for liquid junction potentials

*, electrophysiological estimation; NA, not applicable; #, function does not exhibit voltage-dependency in activation rate and is held constant at 0.1 ms

## Results

We used Pitx3-GFP mice that express GFP selectively in midbrain dopamine neurons (Zhao et al. 2004), to conduct targeted whole-cell recordings from vlPAG/DRN DA neurons in acute brain slices, an approach that we have previously described (Dougalis et al. 2012). Under these conditions, most DA neurons in the vlPAG/DRN fire action potentials spontaneously (Dougalis et al. 2012).

### Hyperpolarization-activated cation current (I_H_)

**Fig. 1:**
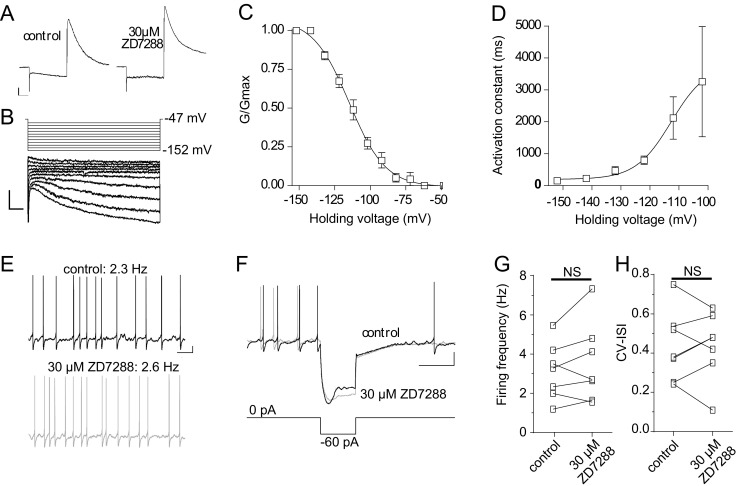
Hyperpolarization-activated cation current (I_H_ current). **a**. Representative voltage-clamp electrophysiological traces from a vlPAG/DRN DA neuron showing the expression of a hyperpolarization-activated inward current (I_H_) through a single hyperpolarizing step from −62 to −152 mV (500 ms duration). This current was sensitive to ZD 7288 (30 μM), a reputed specific blocker of the I_H_ conductance (scale bars, 50 pA and 125 ms). **b**. Electrophysiological traces of I_H_ current activation taken from a vlPAG/DRN DA neuron. A series of voltage steps in 10 mV increments was delivered (holding potential −47 mV) from −62 to −152 mV for 1 s (scale bars, 25 pA and 100 ms). **c**. Average steady-state activation curve (G/G_max_) for the I_H_ conductance for vlPAG/DRN neurons recorded through a series of hyperpolarizing voltage steps from −52 mV to −152 in 10 mV increments as shown in B (*n* = 6). Normalised conductance plots were fitted with a single Boltzmann function to calculate mean V_50_ and slope values (activation,-114 mV; slope, −12.7; *n* = 6). **d**. Voltage-dependence of I_H_ current activation time constant. Activation time constant was voltage-dependent and become faster at more positive potentials (mean τ_act_ of 151 ms at −152 mV). Data were plotted against holding voltage and fitted with a single Boltzmann function to calculate mean V_50_ (−112.7 mV) and slope (6.1) values (*n* = 6). **e**. Representative current-clamp electrophysiological traces taken from a vlPAG/ DRN DA neuron showing the effect of ZD 7288 (30 μM) on firing frequency. The I_H_ blocker did not have any effects on the frequency of firing on this cell (control, 2.3 Hz and in ZD 7288, 2.6 Hz) (scale bars, 10 mV and 1 s). **f**. Representative current-clamp electrophysiological traces from the neuron in E showing the effect of ZD 7288 (30 μM) on the hyperpolarization-induced voltage-sag. The I_H_ blocker blocked the voltage-sag in response to hyperpolarizing current injection (−60 pA, scale bars, 20 mV and 1 s). **g**. Bar chart comparison of average firing frequency (Hz) before and after application of ZD 7288 (30 μM) as shown in E for eight vlPAG/DRN DA neurons. ZD 7288 did not induce any significant change in the firing frequency of vlPAG/DRN DA neurons (mean firing frequency in control, 3.1 Hz; in ZD 7288, 3.5 Hz, *n* = 7, paired t-test; NS, not significant). H. Bar chart comparison of average CV-ISI before and after application of ZD 7288 (30 μM) as shown in E for seven DA neurons. ZD 7288 did not induce any significant change in CV-ISI of vlPAG/DRN DA neurons (mean CV-ISI in control, 0.46; in ZD 7288, 0.44, *n* = 7, paired t-test; NS, not significant)

Midbrain DA neurons express a hyperpolarization-activated inward current (I_H_) whose blockade can decrease pacemaker frequency of some, but not all, SNc DA neurons (see Neuhoff et al. 2002; Seutin et al. 2001; Khaliq and Bean 2010). The I_H_ current recorded in vlPAG/DRN DA neurons was sensitive to the specific I_H_ current blocker ZD7288 (30 μM) which completely ablated the inward currents recorded under a hyperpolarizing pulse from −62 to −132 mV in voltage-clamp (87 ± 4% current block, *n* = 6, from three mice, Fig. 1a). A series of hyperpolarizing pulses from −52 mV to −152 mV for 1 s in the presence of TTX (*n* = 6, three mice, Fig. 1b) resulted in I_H_ current steady-state activation values for V_50_ and slope of −114.7 ± 1.7 mV and −12.7 ± 1.6 respectively (Fig. 1c). I_H_ current activation time constant was voltage-dependent becoming faster at more positive potentials (2118 ± 664 ms at −102 mV and 151.0 ± 24 ms at −152 mV, *n* = 6, *P* < 0.001, paired t-test) with V_50_ and slope values of −112.7 ± 7.8 mV and 6.1 ± 5.1 respectively (Fig. 1d). Maximal I_H_ conductance was calculated to be 0.56 ± 0.01 nS at −152 mV (*n* = 6). Results quantifying I_H_ current steady-state activation and activation kinetics *via* a current subtraction protocol (before and after addition of ZD7288) did not differ from results obtained using the simplified activation voltage-clamp protocol used above and thus the subtraction protocol was not routinely used for measurements of I_H_ current kinetics of vlPAG/DRN DA neurons (data not shown). To evaluate the impact of I_H_ current blockade on autorhythmicity and pacemaker frequency of DA neurons, ZD7288 (30 μM) was perfused under current-clamp conditions while monitoring spontaneous firing in the presence of synaptic blockers for glutamatergic and GABAergic transmission (see methods). The I_H_ current blocker, did not affect firing frequency of DA neurons (firing frequency in control, 3.1 ± 0.5 Hz; in ZD 7288, 3.5 ± 0.8 Hz, *n* = 7 from four mice, *P* > 0.05, paired t-test, Fig. 1e, g) or firing regularity (CV-ISI in control, 0.46 ± 0.07; in ZD7228, 0.44 ± 0.07, *n* = 7 from four mice, *P* > 0.05, paired t-test, Fig. 1h) despite eliminating the I_H_ mediated voltage-sag under current-clamp induced by hyperpolarizing current injections (−60 pA for 1 s, Fig. 1f). For comparative purposes we also recorded I_H_ current in SNc and VTA DA neurons (three mice). As expected, both midbrain nuclei responded with an inward current upon a series of hyperpolarizing voltage steps (from −62 to −132 mV in 10 mV increments, online resource 1A) albeit with evidently differential current steady-state activation midpoint (online resource 1B) and kinetics (online resource 1C). SNc neurons had more positive V_50_ values (SNc, −92 ± 2.3 mV, *n* = 6–7 for each set, *P* < 0.01 to both VTA and vlPAG/DRN, ANOVA with Tukey’s post-hoc test) for I_H_ current activation and a significantly faster activation time constant (at −132 mV, SNc, 114 ± 14 ms, *n* = 6–7 for each set, *P* < 0.05 to both VTA and vlPAG/DRN, ANOVA with Tukey’s post-hoc test) than either VTA (−121 ± 5.6 mV and 397 ± 84 ms respectively) or vlPAG/DRN (−121 ± 3.5 mV and 260 ± 34 ms respectively) that exhibited comparable profiles without any significant differences between them in either parameter (VTA *vs* vlPAG/DRN, *P* > 0.05 for both comparisons, unpaired t-test). Also no differences were detected in the slope of the activation curve (slope, SNc, −11.8 ± 2.6; VTA, −12.0 ± 2.8; vlPAG/DRN, −13.0 ± 1.8, *n* = 6–7 for each set, *P* > 0.05, ANOVA with Tukey’s post-hoc test).

### A-type (I_A_) transient potassium currents

**Fig. 2:**
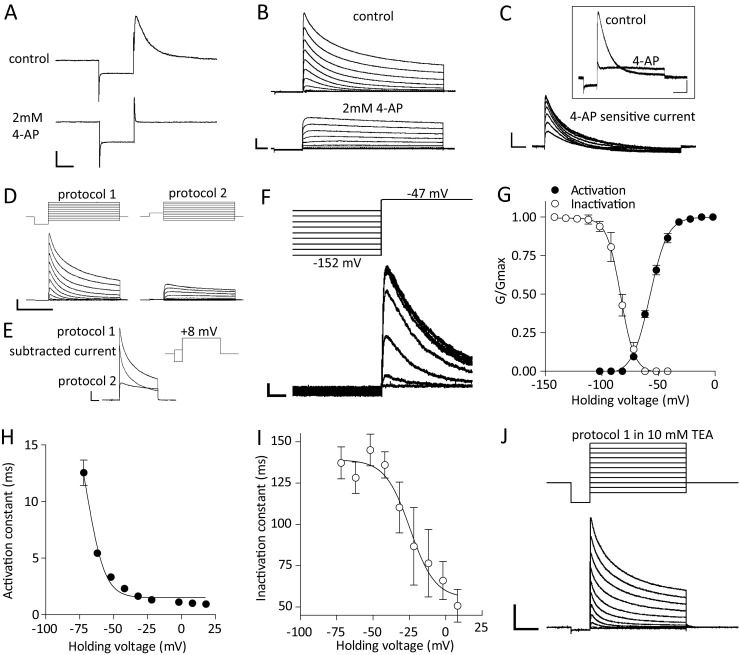
A-type (I_A_) potassium current. **a**. Electrophysiological traces of voltage-clamp recordings (single step from −62 to −102 mV for 250 ms) from a vlPAG/DRN DA neuron before and after addition of 4-aminopyridine (4-AP, 2 mM) in the presence of TTX (1 μM). The outward tail current elicited after the end of the hyperpolarizing step (return to the holding potential of −62 mV) was completely blocked by the I_A_ potassium current blocker 4-AP (scale bars, 50 pA and 100 ms). **b**. Electrophysiological traces of voltage-clamp recordings (prepulse step of 250 ms from −62 to −112 mV before a series of test steps of 1 s in duration from −92 to +8 mV in 10 mV increments) from a vlPAG/DRN DA neuron before and after addition of 4-aminopyridine (4-AP, 2 mM). The transient outward currents elicited upon depolarization were blocked by the I_A_ potassium current blocker 4-AP leaving a residual sustained current (scale bars, 500 pA and 100 ms). **c**. Overlaid electrophysiological traces from experiment shown in B depicting transient outward currents (test pulse to −52 mV) before and after addition of 4-aminopyridine (4-AP, 2 mM, inset, scale bars 100 pA and 200 ms) accompanied by an overlay of the digitally subtracted 4-AP sensitive current (bottom traces, scale bars 200 pA and 200 ms) for the whole series of steps shown in B (depicted test steps from −72 to −12 mV). Note that the currents before and after 4-AP ‘cross’ signifying that 4-AP blocks the I_A_ transient outward current but also increases a background conductance. **d**. Activation of transient and sustained outward potassium currents recorded using a series of depolarizing test pulses (1 s duration, from −92 to +8 mV, 10 mV increments) *via* two different single prepulses (250 ms duration from −72 to −112 mV and −72 to −52 mV, protocols 1 and 2 respectively) in the presence of TTX (1 μM). Protocol 1 recruited fast activating transient and sustained outward currents using a prepulse to −112 mV to facilitate I_A_ current’s recovery from inactivation, while protocol 2 recruited only slowly activating sustained outward currents using a prepulse to −52 mV to inactivate I_A_ current (scale bars, 500 pA and 500 ms). Digital subtraction of the responses (protocol 1 minus protocol 2) was used to isolate the transient I_A_ potassium conductance from background sustained currents. **e**. Overlaid electrophysiological traces obtained *via* voltage protocols 1 and 2 (taken from D). Responses for each protocol together with the resultant digitally subtracted I_A_ current (protocol 1 minus protocol 2) are shown at a single test voltage step of +8 mV for clarity (scale bars, 500 pA and 200 ms). **f**. Inactivation of transient I_A_ outward potassium current recorded using a series of depolarizing prepulses (1 s duration, from −152 to −52 mV) to differentially recover I_A_ current followed by a test pulse (to −47 mV) to record its activation as a function of the prepulse potential (scale bars, 100 pA and 50 ms). **g**. Average steady-state activation and inactivation curves (G/G_max_ against holding voltage) for the subtracted (protocol 1 minus protocol 2) I_A_ current. Normalised conductance plots were fitted with a single Boltzmann function to calculate mean V_50_ (activation,-57.5 mV; inactivation, −87.4 mV) and slope (activation, 7.8; inactivation, −6.1) values (*n* = 6). **h**. Voltage-dependence of I_A_ current activation time constant. Subtracted I_A_ currents were fitted with a single exponential function (start to peak) at each test holding voltage to calculate the activation time constant. Activation time constant appeared voltage-dependent and became faster at more positive potentials (mean τ_act_ of 0.95 ms at +8 mV). Data were plotted against holding voltage and fitted with a single Boltzmann function to calculate mean V_50_ (−68.8 mV) and slope (−5.0) values (*n* = 6). **i**. Voltage-dependence of I_A_ current inactivation time constant. Subtracted I_A_ currents were fitted with a single exponential function (peak to end) at each test holding voltage to calculate the inactivation time constant. Inactivation time constant appeared voltage-dependent and became faster at more positive potentials (mean τ_ina_ of 50.8 ms at +8 mV). Data were plotted against holding voltage and fitted with a single Boltzmann function to calculate mean V_50_ (−24.6 mV) and slope (−8.6) values (*n* = 6). **j**. Typical electrophysiological traces obtained *via* activation voltage protocol 1 (as shown in D) in the presence of 10 mM TEA to block delayed rectifier currents as an alternative method to obtain I_A_ currents without digital subtraction between two protocols (*e.g.* Silva et al. 1990). I_A_ currents obtained exhibited analogous behaviour to that obtained *via* the two protocol subtraction and the 4-AP subtraction methods (see also online resource 2; scale bars, 500 pA and 250 ms)

One of the hallmarks of the electrophysiological behavior of DA neurons in the vlPAG/DRN brain slice is the pronounced outward tail current recorded at subthreshold potential (−60 to −50 mV) following a hyperpolarizing pulse (beyond −85 mV) under voltage-clamp (Fig. 2a). The amplitude of the outward transient tail conductance after a hyperpolarizing step was suppressed (90.1 ± 2.4% at −62 mV, *n* = 6 from three mice) by 2–3 mM 4-aminopyridine (4-AP) indicating that it represents an A-type transient outward potassium conductance (I_A_ current, Fig. 2a, b). Subtraction of the 4-AP sensitive current lead to the typical ‘current crossing’ (Fig. 2c) as reported previously by others (Khaliq and Bean 2008) suggesting that 4-AP also increases some background conductance while blocking the I_A_ current (and potentially blocking a fast delayed rectifier conductance in some neurons, see Itri et al. 2005) making its kinetic description difficult. An alternative method to examine I_A_ current steady-state activation is using digital current subtraction between two different activation protocols in the presence of TTX (1 μM) as reported previously (Koyama and Appel 2006a). Responses to protocol one (Fig. 2d) activated transient and sustained outward currents while responses to protocol two activated slowly-activating sustained outward currents only (reminiscent of delayed rectifier potassium currents, Fig. 2d). Subtraction of the responses (protocol 1 minus protocol 2) resulted in isolation of the I_A_ current (Fig. 2e). Steady-state inactivation was studied using a single standard inactivation protocol without digital current subtraction since the peak of the I_A_ current at the test voltage studied (−47 mV) was not contaminated by slowly activating outward currents (Fig. 2f). Maximal I_A_ conductance was calculated to be 10.0 ± 1.1 nS at +8 mV (*n* = 6, from four mice). The V_50_ value for steady-state activation was −57.5 ± 0.5 mV with a slope of 7.7 ± 0.4, while the corresponding values of V_50_ for steady-state inactivation and its slope were −87.4 ± 0.8 mV and −6.1 ± 0.8 respectively (*n* = 6, Fig. 2g). Activation time constant for I_A_ current was voltage-dependent becoming faster at more positive potentials (5.45 ± 0.27 ms at −62 mV and 0.95 ± 0.06 ms at +8 mV, *n* = 6, *P* < 0.001, paired t-test) with a V_50_ and slope values of −68.8 ± 0.7 mV and −5.0 ± 0.7 respectively (Fig. 2h). Inactivation kinetics were also voltage-dependent with faster inactivation at more positive potentials (128.3 ± 9.5 ms at −62 mV and 50.8 ± 10.0 ms at +8 mV, *n* = 6, *P* < 0.01, paired t-test) with V_50_ and slope values of −24.6 ± 5.7 mV and −8.6 ± 5.3 respectively (Fig. 2i). A second, voltage-independent, slow inactivation time constant (τ_slow_) could be fitted to I_A_ currents recorded more positively than −30 mV and contributed to approximately 35% and 50% of the maximal I_A_ current amplitude recorded at −22 and +8 mV respectively (357 ± 53 ms at −22 mV and 562 ± 106 ms at +8 mV, *n* = 6, *P* > 0.05, paired t-test, online resource 2A). We did not further investigate the origin of this slow conductance but we noted interestingly that 4-AP subtracted currents (as shown in Fig. 2c) not only exhibited a similar average activation steady-state curve (V_50_ of −61.4 ± 0.9 and slope of 7.1 ± 0.8, *n* = 3, online resource 2B) to currents recorded here with the two protocol current subtraction method, but more importantly, 4-AP sensitive currents also exhibited consistently a bi-exponential decay with commensurate, voltage-dependent, fast and slow inactivation time constants that differentially contributed to current amplitude at a range of holding voltages (online resource 2C, D). To examine further the properties of I_A_ currents and to compare our results obtained with the methods described above we recruited I_A_ currents *via* a single activation protocol (protocol 1) in the presence of 10 mM TEA (as described previously by Silva et al. 1990) a method that does not rely on digital current subtraction. This resulted in recording I_A_ currents (Fig. 2j) with analogous average activation steady-state V_50_ of −55.6 ± 1.0 mV, but shallower activation slope of 15.1 ± 1.1 (*n* = 5, from three mice, online resource 2E), to the two protocol subtraction method and the 4-AP-sensitive I_A_ currents. Interestingly, the single protocol I_A_ current isolation in TEA also resulted in a bi-exponential inactivation of recorded I_A_ currents with differential contributions to current amplitude at different voltages (online resource 2F, G). However, unlike the previous methods, inactivation kinetics under these circumstances did not exhibit strong voltage dependence at positive potentials (online resource 2F). Both 4-AP-sensitive I_A_ currents and I_A_ currents isolated in TEA exhibited commensurate voltage-dependent activation kinetics (faster at more positive potentials) in good agreement with the two protocol current subtraction method (data not shown).

### *Delayed rectifier* (I_Kdr_) *sustained potassium currents*

**Fig. 3:**
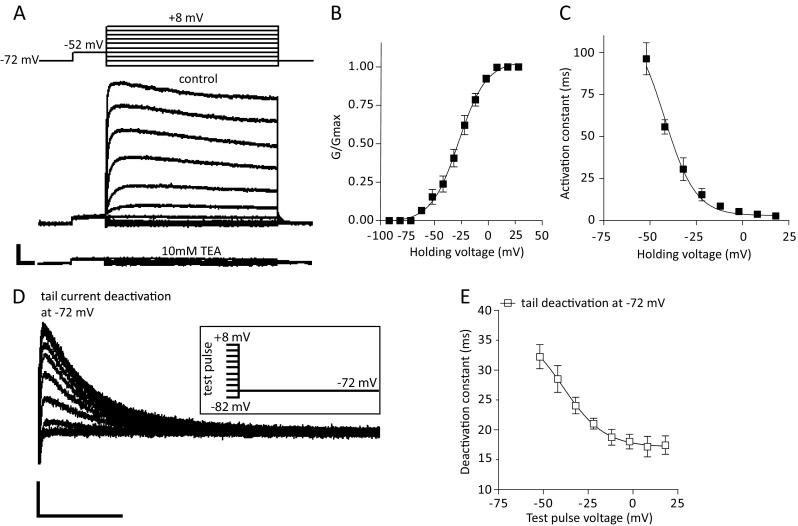
Delayed rectifier (I_Kdr_) potassium current. **a**. Top: Representative electrophysiological traces of slowly-activating, sustained, outward potassium currents (I_Kdr_) recorded using a prepulse (from-72 mV to −52 mV for 250 ms) before the delivery of series of depolarizing test steps (1 s duration, from −72 to +8 mV, 10 mV increments) in the presence of 1 μM TTX (scale bars, 100 pA, 100 ms). Bottom: Sustained outward currents and tail currents were sensitive to TEA (10 mM). **b**. Average steady-state activation curve (G/G_max_) for the slowly-activating, sustained potassium current. Normalised conductance plots were fitted with a single Boltzmann function to calculate mean V_50_ (−26.9 mV) and slope (13.4) values (*n* = 6). **c**. Voltage-dependence of I_Kdr_ current activation time constant. Currents were fitted with a single exponential function (start to peak) at each test holding voltage to calculate the activation time constant. Activation time constant appeared voltage-dependent and became faster at more positive potentials (mean τ_act_ of 2.8 ms at +8 mV). Data were plotted against holding voltage and fitted with a single Boltzmann function to calculate mean V_50_ (−38.4 mV) and slope (−6.9) values (*n* = 6). **d**. Overlaid electrophysiological traces of deactivating tail currents recorded at −72 mV. Tail currents were evaluated at −72 mV following a prepulse (to −52 mV) and a series of depolarizing test voltage steps (protocol as in A, shown truncated in the inset for clarity) that recruited the I_kdr_ current (scale bars, 50 pA, 25 ms). **e**. I_Kdr_ tail current deactivation time constant recorded at −72 mV as a function of a series of depolarizing test holding voltages. Tail currents were fitted with a single exponential function (peak to end) and results are plotted against test holding voltage before returning to −72 mV. Deactivation time constant appeared dependent on test voltage and became faster at −72 mV when neurons were returned to that potential from more positive test potentials (mean τ_deact_ of 32 ms following a test pulse at −52 mV and 17 ms following a test pulse at +8 mV). Data were plotted against test holding voltage and fitted with a single Boltzmann function to calculate mean V_50_ (−38.9 mV) and slope (−11.5) values (*n* = 6)

Sustained, non-inactivating/slowly inactivating, outward currents reminiscent of the delayed rectifier I_Kdr_ were isolated using KGlu based-internal solution in the presence of TTX (1 μM) *via* a standard voltage-clamp activation protocol as reported previously (Silva et al. 1990). Currents recruited were slowly activating, largely non-inactivating during the depolarizing 1 s step, exhibiting a fast deactivation upon return to negative holding potentials and sensitivity to tetraethylammonium (TEA, 10 mM) suggesting the presence of a delayed rectifier current I_Kdr_ in vlPAG/DRN DA neurons (Fig. 3a). Maximal I_Kdr_ conductance was calculated to be 7.0 ± 0.6 nS at +8 mV (*n* = 6, from four mice). The V_50_ value for steady-state activation was −26.9 ± 1.7 mV with a slope of 13.4 ± 1.8 (*n* = 6, Fig. 3b). Activation time constant for I_Kdr_ current was voltage-dependent becoming faster at more positive potentials (98.1 ± 7.7 ms at −52 mV and 2.8 ± 0.2 ms at +8 mV, *n* = 6, *P* < 0.001, paired t-test) with V_50_ and slope of values of −38.4 ± 1.0 mV and −6.8 ± 0.9 respectively (Fig. 3c). The I_Kdr_ current did not exhibit any appreciable inactivation even on long 10 s pulses at potentials up to −20 mV. We also monitored the deactivation of the I_Kdr_ tail current upon return to a single potential of −72 mV following the series of test potentials used to recruit the I_Kdr_ current as described above (Fig. 2d). Deactivation of the I_Kdr_ tail currents at −72 mV was sensitive to the test voltage potential prior to return to −72 mV and became faster at more positive test holding voltages (32.3 ± 2.0 ms following return from −52 mV and 17.2 ± 1.7 ms following return from +8 mV, *n* = 6, *P* < 0.001, paired t-test) with V_50_ and slope of values of −38.9 ± 12 mV and −11.5 ± 6.7 respectively (Fig. 3e). Activation of delayed rectifier-type currents is usually associated with Kv2.1 and/or Kv2.2 families of voltage-gated potassium channels in sympathetic neurons (Malin and Nerbonne 2002) and SNc DA dissociated neurons (Kimm et al. 2015). A K_V_7 (KCNQ)-mediated current (also known as the M-type current, I_M_) has been has been described in VTA neurons (Koyama and Appel 2006b). The I_M_ potassium current is a slowly activating, non-inactivating current with sensitivity to TEA (through Kv7.2 subunits, Hadley et al. 2000) and activation that partially overlaps with that of delayed rectifier currents (Brown and Adams 1980; see also review, Brown and Passmore 2009). To examine the possibility of the presence of an I_M_ current in vlPAG/DRN DA neurons we used a modified deactivation tail protocol (Koyama and Appel 2006b) where neurons were held at −72 mV and given an 1 s prepulse to −32 mV to activate the I_M_ current before stepped to −42 to −72 mV 1 s to record the resultant I_M_ current deactivation tail in the presence of TTX (1 μM) (online resource 2A, B). We recorded I_M_ current relaxations (online resource 3C) with similar properties to that reported previously for VTA neurons (Koyama and Appel 2006b). These were sensitive to the KCNQ blocker XE991 (30 μM, online resource 3A, B, D). Moreover, XE991 partially inhibited the depolarization-induced sustained outward currents at −32 mV (67 ± 7% of control, *n* = 4, online resource 3D) suggesting that depolarizing pulses recruit effectively a large component of sustained outward current that is carried forward by the KCNQ family of voltage-gated potassium channels. Finally, perfusion with XE991 (30 μM) induced a depolarizing current in voltage clamp at −70 mV (control holding current at −70 mV, −18.2 ± 2.1 pA, in XE991, 27.2 ± 1.5 pA, *n* = 4, *P* < 0.05, paired t-test, data not shown).

### Low and high-voltage activated (LVA and HVA) calcium currents (I_CaLVA_ and I_CaHVA_)

**Fig. 4:**
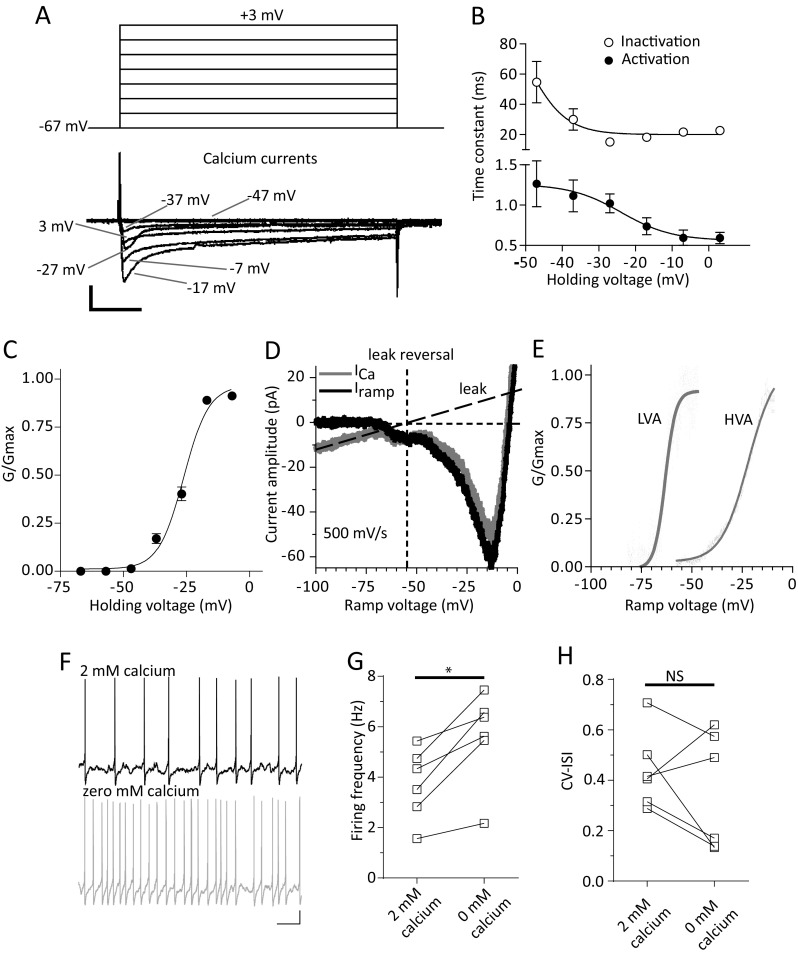
Calcium currents (I_Ca_). **a**. Typical electrophysiological traces of inward calcium currents recorded using a series of depolarizing test steps (250 ms duration, holding potential −67 mV, pulses from −67 to +3 mV, 10 mV increments) in the presence of TTX, 4-AP and TEA (see methods). Note that the calcium currents inactivate during the long test step (scale bars, 50 pA and 50 ms). **b**. Voltage-dependence of activation and inactivation time constants (τ_act_ and τ_ina_) of recorded calcium currents (as shown in A, *n* = 4). To measure τ_act_ and τ_ina_ time constants calcium currents were fitted a single exponential function (start to peak and peak to end respectively) at each holding potential. Both τ_act_ and τ_ina_ were voltage-dependent, becoming faster at more positive potentials (mean τ_act_ and τ_ina_ at −47 mV, 1.27 ms and 114 ms; at −17 mV, 0.62 ms and 16.7 ms respectively). Plotted data were then fitted with a single Boltzmann function to calculate mean V_50_ (activation, −23.8 mV; inactivation, −39.0 mV) and slope (LVA, −6.9; HVA, −2.6) values (*n* = 4). **c**. Average steady-state activation curve (G/G_max_) for calcium currents recorded using a series of depolarising pulses (as shown in A). Normalised conductance plot was fitted with a single Boltzmann function to calculate mean V_50_ (−26.1 mV) and slope (5.0) values for steady state activation (*n* = 4). **d**. Fast voltage-ramp (500 mV/s, −107 to +13 mV) depicting the activation of a calcium currents. Two distinct peaks were identified (both sensitive to cadmium, not shown) that indicate that LVA and HVA calcium currents are expressed on this vlPAG/DRN neuron. Leak subtracted current (dotted line) revealed that LVA calcium currents peaked at around −60 mV and HVA calcium currents peaked at around −15 mV. Leak current reversal under these conditions was −55 mV. **e**. Steady-state activation curves (G/G_max_) for LVA and HVA calcium currents for the neuron shown in D. Normalised conductance plots were fitted with a single Boltzmann function to calculate V_50_ (LVA, −63.2 mV; HVA, −22.1 mV) and slope (LVA, 2.6; HVA, 6.7) values for steady-state activation. **f**. Representative electrophysiological traces recorded in current-clamp mode in normal (2 mM) or zero calcium (see methods) aCSF. Substitution of magnesium with calcium increased spontaneous firing of vlPAG/DRN DA neurons without affecting the firing rate regularity (scale bars, 10 mV and 2 s). **g**. Bar chart comparison of mean firing frequency before and after replacement of 2 mM calcium for magnesium as shown in F for seven vlPAG/DRN DA neurons. This replacement caused a statistically significant change in firing rate (mean firing frequency in 2 mM calcium, 3.3 Hz; in zero mM calcium, 4.9 Hz, *n* = 6, *P < 0.05, paired t-test). **h**. Bar chart comparison of CV-ISI before and after replacement of 2 mM calcium for magnesium as shown in F for seven vlPAG/DRN DA neurons. This replacement did not cause a statistically significant change in CV-ISI (mean CV-ISI in 2 mM calcium, 0.66; in zero mM calcium, 0.40, *n* = 6, *P* > 0.05, paired t-test, NS, not significant)

Calcium currents play an important role in neuronal autorhythmicity and pacemaking in DA midbrain neurons (Wolfart and Roeper 2002; Puopolo et al. 2007; Khaliq and Bean 2010). We have isolated and recorded calcium currents on vlPAG/DRN DA neurons using standard activation protocols in the presence of blockers of potassium and sodium conductances (Brevi et al. 2001; Pignatelli et al. 2005). Calcium currents exhibited a characteristic inward current-voltage relationship with the maximal amplitude (I_max_) occurring around −15 to −10 mV (Fig. 4a) and were fully sensitive to cadmium (200 μM, *n* = 4, from three mice, data not shown). They exhibited a fast, voltage-dependent, activation time constant (τ_act_, 1.27 ± 0.28 ms at −47 mV and 0.62 ± 0.06 ms at −17 mV, *n* = 4, *P* < 0.05, paired t-test, Fig. 4b) and inactivated in a voltage-dependent manner during the 250 ms long steps used (inactivation time constant τ_ina_, 114 ± 28 ms at −47 mV and 16.7 ± 1.7 ms at −17 mV, *n* = 4, *P* < 0.05, paired t-test, Fig. 4b). The corresponding mean V_50_ values for the voltage dependency of the activation and inactivation time constants were −23.9 ± 4.3 mV and −39.0 ± 2.1 mV with a slope of −6.9 ± 3.8 and −2.6 ± 2.1 respectively (*n* = 4). Mean steady-state activation V_50_ for the recorded calcium currents using a series of voltage steps was −26.1 ± 0.7 mV with a slope value of 5.0 ± 0.6 (*n* = 4, Fig. 4c). Using fast voltage-ramps (500 mV/s, from −107 mV to +53 mV) as an alternative way of describing background calcium currents we found that we could successfully discriminate in a subset of vlPAG/DRN DA neurons (*n* = 3 of 7 neurons tested) two peaks of different amplitude (fully sensitive to cadmium), a small one peaking at around −60 to −50 mV and a larger one peaking at around −20 to −10 mV (Fig. 4d, e). Analysis of the voltage ramps suggested that LVA calcium currents had a mean steady-state activation V_50_ of −61.2 ± 2.1 mV with a slope value of 3.9 ± 0.9 (*n* = 3) while HVA calcium currents exhibited a mean steady-state activation V_50_ of −22.4 ± 1.7 mV with a slope value of 7.1 ± 1.0 (*n* = 3). To test the effects of calcium current abolition on spontaneous firing we replaced calcium ions with magnesium and recorded in current-clamp mode using KGlu-filled electrodes in standard aCSF in the presence of synaptic blockers (*n* = 6 from three mice, Fig. 4f). We found that this replacement did not prevent expression of spontaneous firing and that it significantly accelerated pacemaking frequency (control firing frequency, 3.7 ± 0.6; firing frequency in zero calcium, 5.6 ± 0.8, *n* = 6, *P* < 0.01, paired t-test, Fig. 4g) without affecting firing regularity (mean CV-ISI and median in 2 mM calcium, 0.66 ± 0.29 and 0.42; mean CV-ISI and median in zero mM calcium, 0.44 ± 0.06 and 0.35, *n* = 6, *P* > 0.05, paired t-test, Fig. 4h) suggesting that calcium (and calcium-dependent) currents modulate basal firing frequency but are not responsible in driving spontaneous firing of vlPAG/DRN DA neurons.

### Low and high-voltage activated (LVA and HVA) barium currents (I_BaLVA_ and I_BaHVA_)

**Fig. 5:**
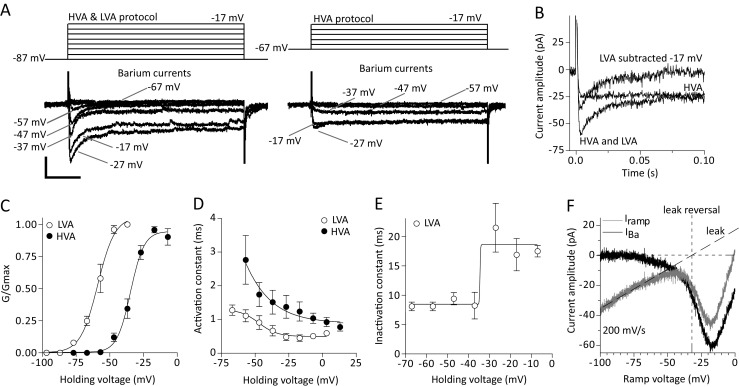
Barium currents (I_Ba_). **a**. Typical electrophysiological traces of inward barium currents recorded using two different protocols (differing in their starting holding voltage) for isolating composite LVA/HVA (left) and HVA only (right) barium currents (see methods). Note the transient barium currents in the LVA/HVA protocol and the non-inactivating barium currents in the HVA protocol during the long test step (scale bars, 25 pA and 50 ms). **b**. Typical electrophysiological traces from HVA/LVA composite and HVA only protocols at a single potential of −17 mV taken from A. Subtraction of the current traces revealed the LVA transient component. **c**. Average steady-state activation curves (G/G_max_) for LVA and HVA barium currents. Normalised conductance plots were fitted with a single Boltzmann function to calculate mean V_50_ (LVA, −58.9 mV; HVA, −34.6 mV) and slope (LVA, 6.3; HVA, 5.1) values for activation (*n* = 6). **d**. Voltage-dependence of barium LVA and HVA current activation time constant. HVA and subtracted LVA currents were fitted a single exponential function (start to peak) at each test holding voltage to calculate τ_act_ for each component. Both HVA and LVA had a τ_act_ that was voltage-dependent becoming faster with more positive potentials (mean LVA and HVA τ_act_ at −47 mV, 0.91 ms and 1.74 ms; at +3 mV, 0.53 ms and 0.78 ms respectively). Plotted data fitted with a single Boltzmann function to calculate mean V_50_ (LVA, −49.2 mV; HVA, −39.7 mV) and slope (LVA, −5.8; HVA, −13.4) values (*n* = 6). **e**. Voltage-dependence of barium LVA current inactivation time constant. Subtracted LVA currents were fitted a single exponential function (peak to end) at each test holding voltage to calculate the inactivation time constant (τ_ina_). Inactivation time constant became slower at more positive voltages (mean τ_ina_ at −47 mV and +3 mV, 8.5 ms and 16.8 ms respectively). Plotted data were fitted with a single Boltzmann function to calculate the mean V_50_ (−34.5 mV) and slope (0.37) values for τ_ina_ (*n* = 6). **f**. Fast voltage-ramp (200 mV/s, −107 to +13 mV) taken from the cell shown in A depicting the activation of a background barium current. Leak subtracted current (dotted line) revealed that barium currents peaked at around −20 mV. Fast voltage-ramps in barium were less efficient than voltage steps in isolating the LVA transient (see also Fig. 4d, e for calcium voltage-ramps). Although we did not observe a well-defined peak at the voltage range of −65 to −55 mV we observed the development of an inward current at such potentials well before the expected start of the activation of HVA current (usually around −35 mV, peaking at −15 to −20 mV). Note the change in the slope of the development of the putative LVA and HVA inward barium currents. Plotted voltage-ramp data were fitted with a single Boltzmann function to calculate the mean V_50_ (−38.9 mV) and slope (6.3) values for barium current steady-state activation. Note that, in the presence of barium, leak current is reversing at around −25 mV which is 30 mV more positive that its reversal when using calcium as the charge carrier suggesting that a barium sensitive leak conductance is operant on vlPAG/DRN DA neurons

Calcium currents are known to undergo two types of inactivation (voltage-dependent and calcium-dependent) in a number of preparations (Giannattasio et al. 1991; Haack and Rosenberg 1994; Catterall 2000; Budde et al. 2002). It is unlikely that the inactivation observed here in calcium during the series of depolarizing voltage steps (starting at −67 mV) is due to a transient low voltage activated (LVA) calcium current as this voltage protocol should not have recruited such currents to any significant degree while the inactivation kinetics of LVA transient currents are reported to be different from the inactivation seen here for calcium currents (Kang and Kitai 1993a; Pignatelli et al. 2005, see also below the inactivation of barium LVA currents). The inactivation of calcium currents recorded here during long steps could be explained by intracellular calcium release inducing calcium current inactivation or could be attributed to the specific properties of a high voltage activated (HVA) calcium conductance (*e.g.* see Keja et al. 1992; Kang and Kitai 1993a, 1993b). To distinguish between these possibilities, we replaced calcium (2 mM) with barium (1 mM) in the extracellular solution to reduce calcium release-induced calcium current inactivation (Hille 2001). We used two protocols to record barium currents, commencing from a different potential that can recruit selectively HVA or a composite of LVA/HVA currents (see Brevi et al. 2001; Hille 2001). Barium replacement resulted in robust non-inactivating HVA currents during long steps (250 ms) used in our study (Fig. 5a) suggesting that calcium release-induced calcium current inactivation is the most likely explanation for the observed inactivation of calcium currents (compare Figs. 4a and 5a). During LVA/HVA composite current protocol recording we noticed the appearance of transient fast activating, fast inactivating barium current (Fig. 5a). These currents were subtracted from HVA currents to reveal the LVA component (Fig. 5b) and both components were individually subjected to kinetic analysis. Maximal conductance (G_max_) for the LVA and HVA calcium component was calculated at 0.65 ± 0.14 nS and 1.06 ± 0.18 nS respectively (*n* = 6, from three mice). Mean steady-state activation V_50_ for LVA and HVA barium currents were −58.9 ± 1.5 mV and −34.6 ± 0.9 mV with slope values of 6.3 ± 1.2 and 5.1 ± 0.8 respectively (*n* = 6, Fig. 5c). Activation time constants were voltage-dependent for both LVA and HVA barium currents, being faster at more positive potentials (τ_act_ at −57 mV and +3 mV, LVA, 1.11 ± 0.18 ms and 0.53 ± 0.09 ms; HVA, 2.77 ± 0.76 ms and 0.78 ± 0.12 ms respectively, *n* = 6, *P* < 0.05 for both comparisons, Fig. 5d). There was also significant difference between LVA and HVA activation at all holding potentials more negative than −7 mV but not at more positive potentials (*n* = 6, *P* < 0.05, paired t-test). Mean activation time constant V_50_ for LVA and HVA barium currents was −49.2 ± 3.4 mV and −39.7 ± 5.1 mV with slope values of −5.8 ± 3.0 and −13.4 ± 5.1 respectively (*n* = 6, Fig. 5d). LVA inactivation time constant exhibited voltage-dependency, being slower at more positive potentials (at −67 mV, 8.5 ± 0.8 ms; at +3 mV, 16.8 ± 1.4 ms, *n* = 6, *P* < 0.05, paired t-test, Fig. 5e), while when fitted with a Boltzmann function in the voltage range it exhibited V_50_ value of −34.5 ± 11.1 mV with slope of 0.37 ± 4.3 (*n* = 6). Fast voltage-ramps (200–500 mV/s, from −107 mV to +13 mV) recruited a barium current peaking at −20 to −30 mV but were less successful in isolating a distinct peak reflecting an LVA component as seen in a some neurons recorded in calcium (*e.g.* Figure 4d). Despite this we found evidence of activation of an inward conductance at potentials more negative than −60 mV (Fig. 5f) of different slope than the activation of the HVA component that is expected to begin at much more positive potentials (starting at around −35 mV). Steady-state activation V_50_ for the background voltage-ramp barium current was −38.9 ± 2.5 mV with slope value of 6.3 ± 1.5 (*n* = 4). Interestingly, leak current reversal during these voltage ramps appeared 30 mV more positive than ramps conducted in calcium (compare ramps in Fig. 4d and 5f) indicating that the leak conductance is probably at least partially carried forward to a degree by potassium ions through barium-sensitive ion channels. Nimodipine (10 μM), a L-type calcium channel blocker, blocked partially the HVA component (30–40% block) evoked by depolarizing steps (from −87 mV in 10 mV increments) without affecting the transient LVA component signifying that at least in part HVA barium currents are carried forward *via* the L-type calcium channel (online resource 4A, B).

### Sodium (I_Na_) currents

**Fig. 6:**
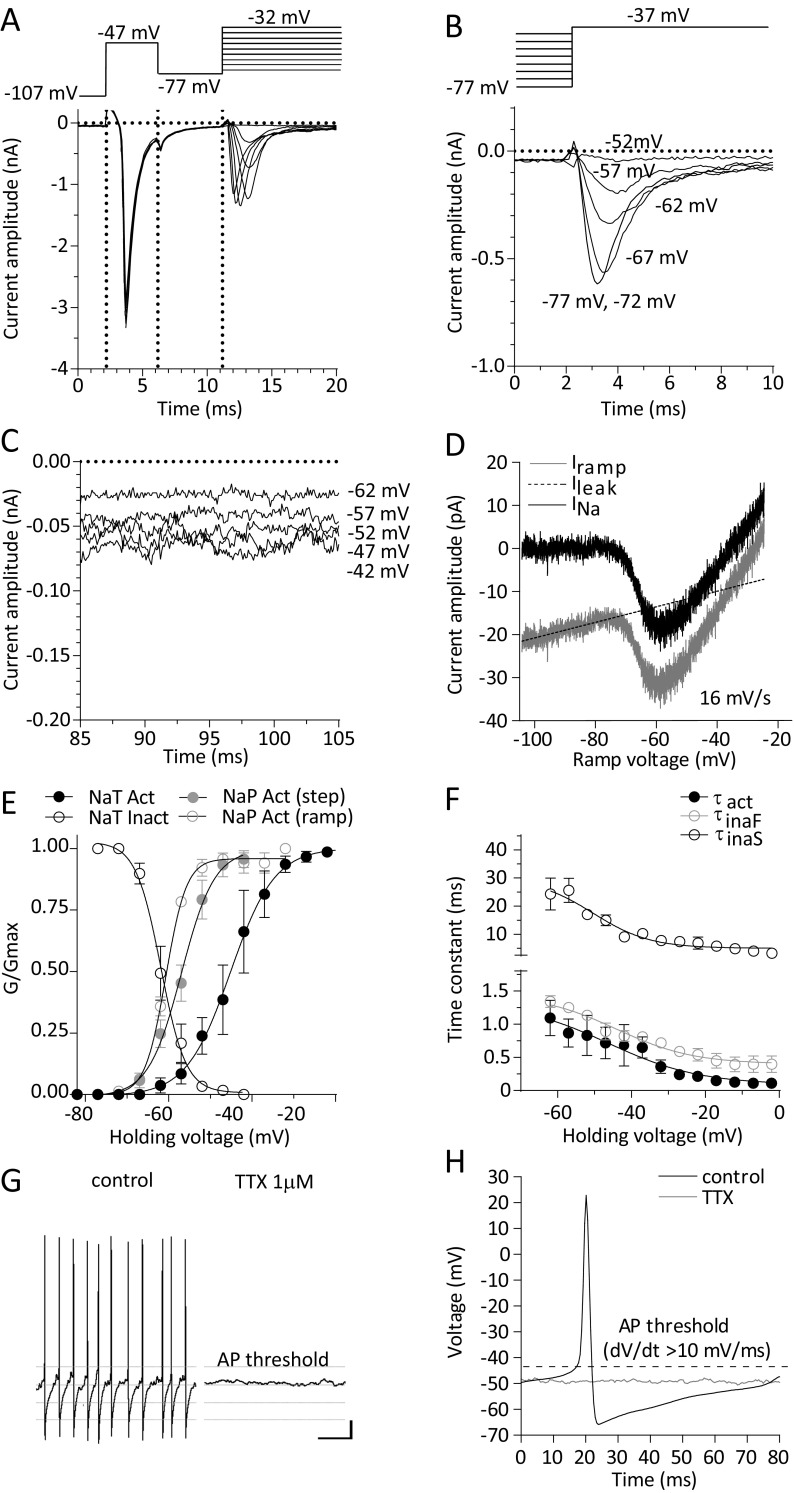
Transient and persistent sodium currents (I_NaT_ and I_NaP_). **a**. Representative average electrophysiological traces depicting the activation of a transient inward sodium current recorded using a protocol that selectively inactivates axonal sodium currents as described previously (Milescu et al. 2010). Neurons were held at −107 mV and a depolarising pulse (4 ms) was delivered to −47 mV to elicit unclamped sodium currents, followed by a brief 5 ms hyperpolarisation to −77 mV to facilitate recovery of somatic (but not axonal) sodium currents before eliciting a series of test pulses in 5 mV increments and of 110 ms in duration to activate somatic sodium currents. Experiments were conducted in the presence of blockers of potassium and calcium conductances. Note the activation of the unclamped distorted sodium current in the initial depolarising 5 ms step and the gradual incremental nature of the sodium currents during the test pulse. **b**. Representative average electrophysiological traces taken from cell shown in A depicting the protocol used to study steady-state inactivation of somatic sodium channels. Holding current and initial depolarisation to activate unclamped sodium currents were identical to the one used for the study of steady-state activation (shown in A) but the subsequent 5 ms hyperpolarising pulse varied from −77 mV to −42 mV while the test pulse for studying the inactivation was set constant to −37 mV. **c**. Representative average electrophysiological traces taken from cell shown in A depicting the persistence of an inward current even after 100 ms of depolarization at different potentials. Test potential are displayed next to each trace. Traces displayed here have not been subtracted for linear leak current. The transient sodium current occurring at the beginning of the traces have been truncated for simplicity. **d**. Slow voltage-ramp (16 mV/s, −107 to +53 mV) depicting the activation of a background persistent sodium current. Leak subtracted current (dotted line) revealed the activation kinetics of the persistent sodium current (I_max_ of 15 pA at −57 mV). **e**. Average steady-state activation/inactivation curves (G/G_max_) for the transient and persistent sodium current (*n* = 6). Persistent sodium current curves have been quantified *via* both a prepulse step protocol shown in A and C (*n* = 6) and through slow voltage ramps as shown in D (*n* = 3). Normalised conductance plots were fitted with a single Boltzmann function to calculate mean steady-state activation V_50_ (transient, −45.2 mV; persistent, −56.6 mV) and its slope (transient, 5.3; persistent, 3.3). Ramp voltage determination of persistent sodium current activation had a mean V_50_ and slope of −60.7 and 2.3 respectively. Mean transient sodium current inactivation V_50_ and slope values were −62.8 mV and −6.4 respectively. **f**. Voltage-dependence of I_NaT_ current activation and inactivation time constants. I_NaT_ currents were fitted a single exponential function (start to peak) and two exponential functions (from peak to end) at each test holding voltage to calculate a single activation (τ_act_) and two (τ_inaF_ and τ_inaS_) inactivation time constants respectively. Activation time constant appeared voltage-dependent becoming faster at more positive potentials (mean τ_act_ of 705 μs at −47 mV and 147 μs at −2 mV). Data were plotted against holding voltage and fitted with a single Boltzmann function to calculate mean V_50_ (−34.4 mV) and slope (−5.6) values (*n* = 6). Inactivation was consistently better fitted with two rather than one exponential functions signifying the presence of a fast and a slow time constant (τ_inaF_ and τ_inaS_). The slow decay constant contributed to about 5% of the maximal sodium current, while both fast and slow inactivation exhibited striking voltage dependency. The τ_inaF_ became faster at more positive potentials (mean τ_inaF_ of 889 μs at −47 mV and 410 μs at −2 mV). Similarly, τ_inaS_ also became faster at more positive potentials (mean τ_inaS_ of 17.4 ms at −47 mV and 4.2 ms at −2 mV). Data were plotted against holding voltage and fitted with a single Boltzmann function to calculate mean V_50_ (−46.4 mV and −50.5 mV) and slope (−11.3 and −7.7) values for the τ_inaF_ and τ_inaS_ components respectively (*n* = 6). **g**. Representative electrophysiological traces recorded under current clamp in standard aCSF using KGlu filled electrodes before and after perfusion of TTX (1 μM). Dotted lines are arranged in 10 mV intervals with top line representing the AP threshold. Note that, TTX caused the development of stable, non-oscillating membrane potential below AP threshold in vlPAG/DRN DA neurons. **h**. Representative electrophysiological trace of averaged (150) action potentials and average membrane potential after TTX superfusion (1 μM) for the cell shown in G. In this cell the membrane potential was on average 7.2 mV more hyperpolarized compared to the average action potential threshold (−43.1 mV)

Transient and background ‘persistent’ sodium currents are operant on VTA and SNc neurons contributing differentially on their autorhythmicity (Puopolo et al. 2007; Khaliq and Bean 2010). We have recorded fast activating/inactivating (I_NaT_) and persistent/non-inactivating inward sodium currents (I_NaP_) through a standard activation depolarization step protocol and voltage-ramps in the presence of cadmium, cesium and TEA to block calcium and potassium currents respectively as reported previously (Pignatelli et al. 2005; Magistretti et al. 2006). Most voltage-clamped vlPAG/DRN DA neurons showed typical signs of problematic space-clamp control upon recordings of the fast transient sodium conductance as reported previously for neurons in brain slices (*e.g.* see Magistretti et al. 2006). Typical signs included distortion of sodium current waveform, clear long latencies to spike (> 4 ms from step onset to peak) and non-graded responses in activation implying activation of unclamped axonic sodium channels. To overcome this problem we have used a modified protocol exploiting a prepulse to selectively inactivate axonal currents and resolve somatic sodium currents as described previously (Milescu et al. 2010). Cells were held at −107 mV and a 4 ms depolarizing pulse to −47 mV was delivered to activate unclamped sodium channels (somatic and axonic, Milescu et al. 2010). Then, a 5 ms hyperpolarizing pulse was delivered to −77 mV to facilitate the fast deactivation of somatic (but not axonal, see Milescu et al. 2010) sodium currents before delivering 110 ms long, incremental 5 mV step depolarizations (up to +13 mV) to record somatic sodium currents. We routinely recorded good quality somatic sodium currents in one every 3–4 neurons at room temperature (20–22 °C) but at a much lower yield of one every 8–10 neurons at higher temperature in line with the problems that occur in electrophysiological recordings of very fast activating currents (Hille 2001; Milescu et al. 2010). At our target temperature of 35 °C, a few vlPAG/DRN DA neurons (*n* = 6, from three mice) exhibited a smooth graded response of somatic sodium current activation in the subthreshold range of −65 to −40 mV were accepted for kinetic analysis (Fig. 6a). Steady-state inactivation was studied by a series of prepulses (−77 to −42 mV for 5 ms, test pulse at −37 mV for 20 ms) delivered after a depolarizing step (−107 to −47 mV for 4 ms) to induce the inactivation of the axonal sodium current as detailed above for the construction of the steady-state activation curve (Fig. 6b). Following the fast transient sodium response, a “persistent”, background, sodium current appeared as an inward non-inactivating/slowly inactivating current upon 110 ms long step depolarizations (Fig. 6c). Both fast and persistent currents were fully sensitive to TTX while we did not detect any consistent residual inward current in the presence of TTX that would imply the activation of a TTX-resistant sodium conductance (*n* = 8, from four mice, data not shown). Slow voltage-ramps (16 mV/s, −107 to +53 mV) confirmed the presence of a background sodium conductance (Fig. 6d) that was 100–150 times smaller in magnitude than the transient sodium conductance. The average G_max_ for the transient sodium component was 16.1 ± 1.2 nS with G_max_ of persistent sodium currents reaching a much smaller value of 0.19 ± 0.04 nS (*n* = 6). Step depolarization and slow voltage-ramp activation protocols resulted in a G_NaP_ that activated 5–10 mV more negative than G_NaT_ (Fig. 6e). Steady-state activation V_50_ values were −45.2 ± 1.4 mV and −56.6 ± 1.3 mV with slope of 5.3 ± 1.3 and 3.3 ± 1.0 for G_NaT_ and G_NaP_ respectively (*n* = 6) whereas the corresponding values for ramp voltage determination of G_NaP_ for V_50_ and slope was −60.7 ± 3.0 mV and 2.3 ± 0.7 (*n* = 3, Fig. 6e). I_NaT_ steady-state inactivation had a V_50_ of −62.8 ± 2.5 and a slope of −6.4 ± 1.7 (*n* = 4, Fig. 6e). I_NaT_ activation time constant (τ_act_) was voltage-dependent becoming faster at more positive potentials (τ_act_ at −47 mV, 704. 5 ± 152.4 μs; at −2 mV, 147.2 ± 17.8 μs, *n* = 6, *P* < 0.05, paired t-test, Fig. 6f). The V_50_ and slope for the τ_act_ was −34.4 ± 4.1 mV and −5.6 ± 3.8 respectively (*n* = 6). The inactivation phase of the sodium current was consistently fitted with two (fast and slow, τ_inaF_ and τ_inaS_) rather than one exponential function. The contribution of the τ_inaS_ exponential component in the inactivation phase of I_NaT_ would account only for <5% of the amplitude of the current for the voltage range studied (up to −2 mV, *n* = 6, data not shown). I_NaT_ inactivation time constants showed similar voltage-dependence (Fig. 6f) with both τ_inaF_ and τ_inaS_ becoming faster at more positive potentials (τ_inaF_ and τ_inaS_ at −47 mV, 888.8 ± 83 μs and 17.4 ± 2.0 ms; at −2 mV, 410.2 ± 80.5 μs and 4.2 ± 0.6 ms respectively, *n* = 6, *P* < 0.05 for both paired comparisons, paired t-test). The corresponding V_50_ values for τ_inaF_ and τ_inaS_ extracted with a first order Boltzmann in the voltage range tested, returned values of -46.4 ± 1.5 mV and −50.5 ± 1.4 mV and slopes of −11.3 ± 1.5 and −7.7 ± 1.4 respectively (*n* = 6). To investigate the impact of sodium channel blockade on spontaneous firing and pacemaker frequency we recorded in current-clamp in control conditions using standard KGlu filled electrodes and superfused the slice with 1 μM TTX (Fig. 6g, h) as reported previously (Khaliq and Bean 2010). We found that after perfusion of TTX the neurons stopped firing and had a stable, non-oscillating, resting membrane potential that was on average − 6.4 ± 0.8 mV more negative than the average action potential threshold (−43.1 ± 1.3 mV) (*n* = 8, from three mice, data not shown). These data suggest that TTX-sensitive sodium currents are important in membrane repolarization to AP threshold and neuron’s spontaneous firing.

### Model DA neuron

#### Validation of *in silico* model DA neuron

**Fig. 7:**
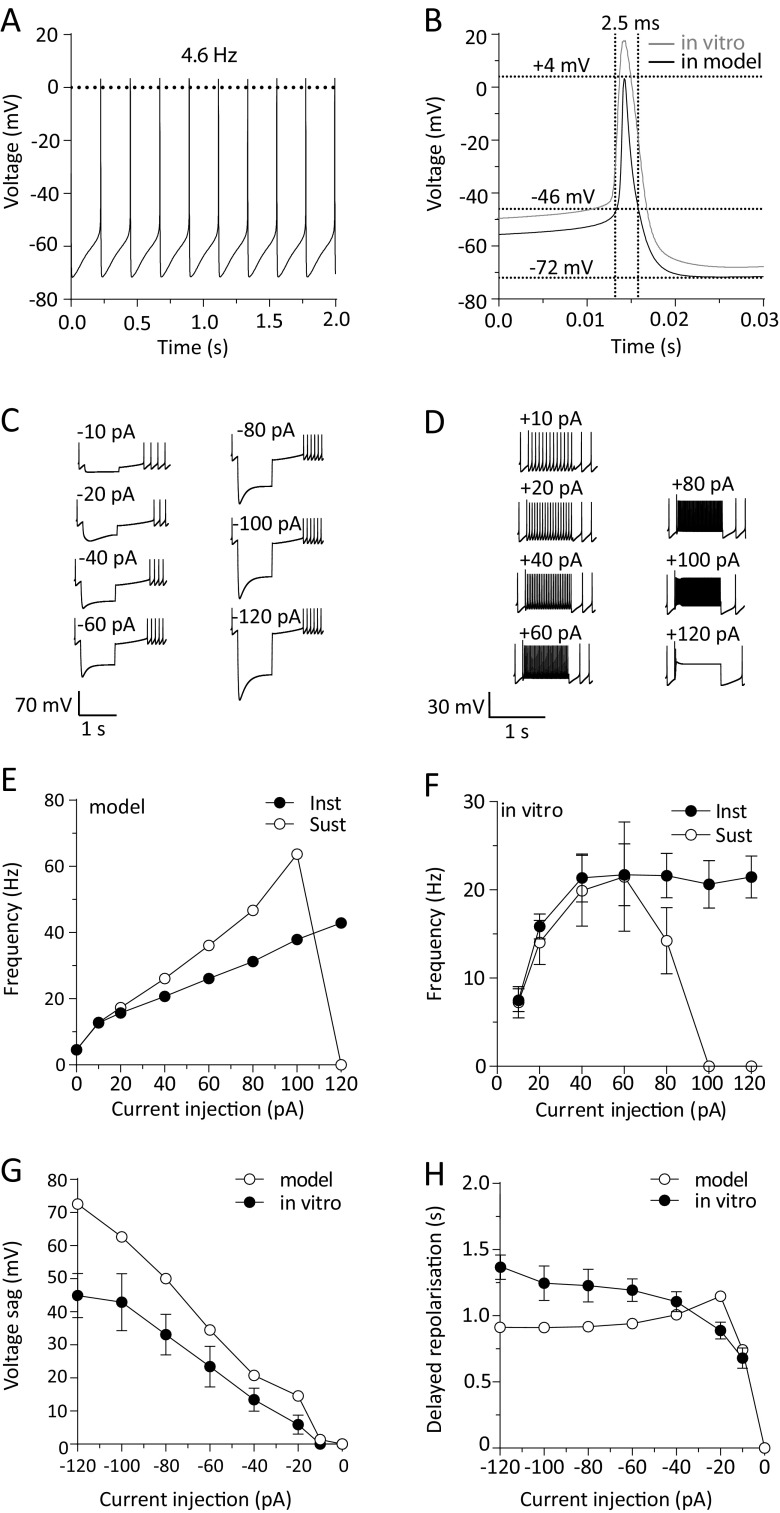
Electrophysiological properties of model DA neuron. **a**. Representative 2 s simulation trace of spontaneous AP firing in model DA neuron (model parameters detailed in Table 1). Model DA neuron fired APs at 4.6 Hz. **b**. Detail of a representative AP in model DA neuron and *in vitro* DA neuron in vlPAG/DRN. Dotted horizontal lines depict the peak of the AP (+ 4 mV), AP threshold (−46 mV) and AHP maximal trough (−72 mV). **c**. Representative simulation traces depicting model DA neuron behaviour following a 1000 ms incremental hyperpolarizing current injection (−10 to −120 pA). Note that, increasing the magnitude of the hyperpolarizing current injection elicited larger voltage-sag responses and delayed repolarisation to firing in model DA neuron. **d**. Representative simulation traces of AP firing in model DA neuron following 1000 ms incremental depolarizing current injection (+10 to +120 pA). Note that, increasing the magnitude of the depolarizing current injection elicited higher frequency of firing and eventually lead to depolarization block (cessation of firing) in model DA neuron. **e**. Input-output relationship for instantaneous and sustained firing frequency following incremental injection of depolarizing current pulses (+10 to +120 pA) in model DA neuron as shown in D. **f**. Input-output relationship for instantaneous and sustained firing frequency following incremental injection of depolarizing current pulses (+10 to +120 pA) in *in vitro* vlPAG/DRN DA neurons (*n* = 5). **g**. Comparative input-output relationship for the hyperpolarization-induced voltage-sag in DA model (as shown in C) and *in vitro* vlPAG/DRN DA neurons (*n* = 8). **h**. Comparative input-output relationship for delayed repolarisation to firing following the termination of hyperpolarizing current pulses in DA model (as shown in C) and *in vitro* vlPAG/DRN DA neurons (*n* = 8)

**Fig. 8:**
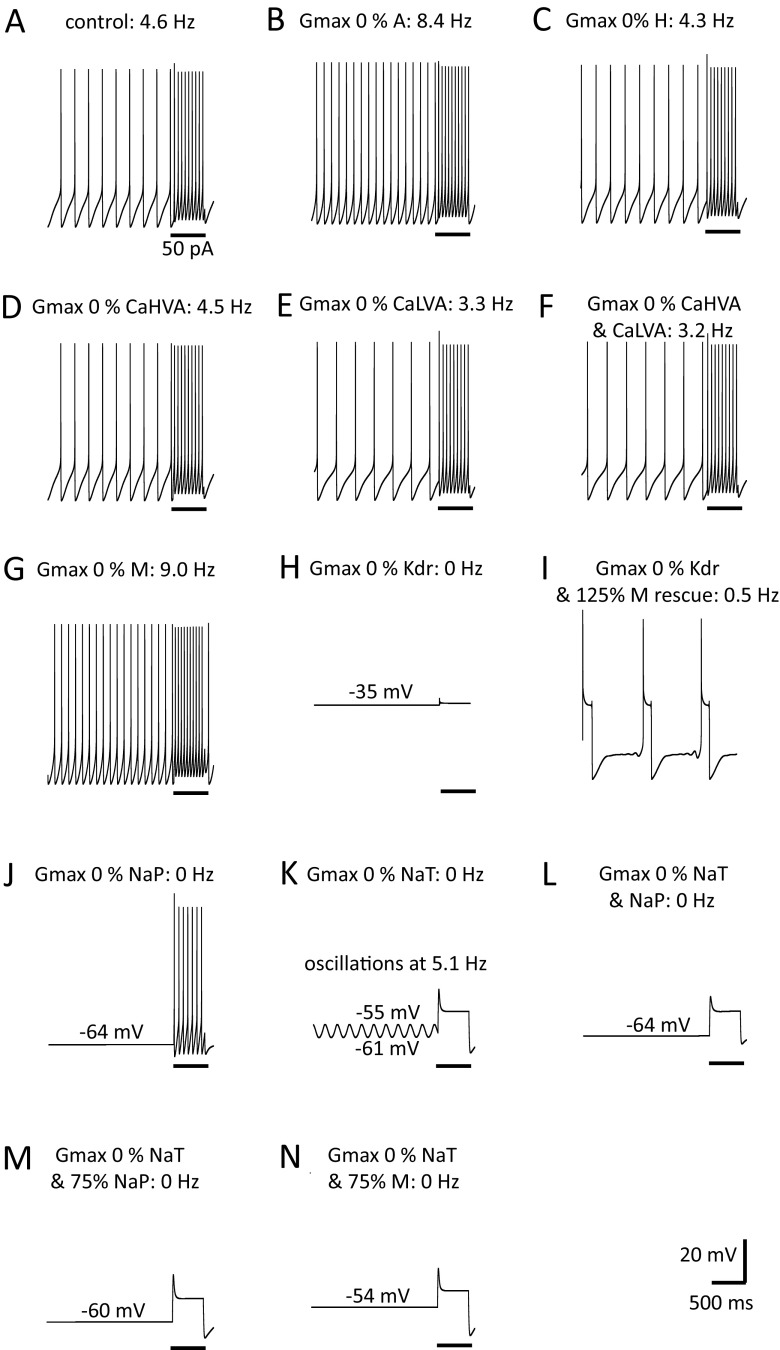
Contribution of individual ionic currents to pacemaker firing frequency and autorhythmicity in model DA neuron. Representative simulation traces depicting the contribution of each individual conductance to AP firing frequency in model DA neuron. Maximal conductance of each current was reduced to 0% of its maximal conductance value (G_max_) and the effects on AP firing frequency were noted. The bar appearing at the end of each trace is the response to a 50 pA depolarising current injection (500 ms). **a**: The model DA neuron fired APs at a basal firing frequency of 4.6 Hz. **b**: I_A_ current elimination lead to a two-fold increase in firing frequency of model DA neuron. **c**: Elimination of the I_H_ current induced a small decrease in firing frequency of model DA neuron. **d**: Elimination of the I_CaHVA_ did not modify firing frequency of model DA neuron. **e**: Elimination of the I_CaLVA_ reduced the frequency of firing of model DA neuron by 25%. **f**: Concomitant elimination of both I_CaHVA_ and I_CaLVA_ calcium conductances did not eliminate spontaneous firing of model DA neuron. **g**: I_M_ current elimination lead to a two-fold increase in firing frequency of model DA neuron. **h**: Elimination of I_Kdr_ current stopped spontaneous firing of model DA neuron. **i**: A small increase of I_M_ current (to 125% of basal G_max_) in the absence of a I_Kdr_ current restored spontaneous firing of model DA neuron. **j**: Elimination of I_NaP_ current lead to the development of a stable non-oscillating membrane potential below AP threshold. However, upon depolarising current injection the model DA neuron still fired APs. **k**: Elimination of I_NaT_ current lead to the development of an oscillating membrane potential below AP threshold (oscillating range, max, −55 mV; min, −61 mV). Upon depolarising current injection the model DA neuron did not fire APs. **l**: Concomitant elimination of both I_NaT_ and I_NaP_ sodium conductances lead to the development of a stable non-oscillating membrane potential (−64 mV) below AP threshold and cessation of spontaneous AP firing. Upon depolarising current injection the model DA neuron did not fire APs. **m**: A small decrease (to 75% of G_max_) in I_NaP_ in the absence of I_NaT_ current stopped the development of a background oscillation in model DA neuron. **n**: A small decrease (to 75% of G_max_) in I_M_ current in the absence of a I_NaT_ current was also successful (as was I_NaP_ alone, see above M) to stop the development of a background oscillation in model DA neuron

**Fig. 9:**
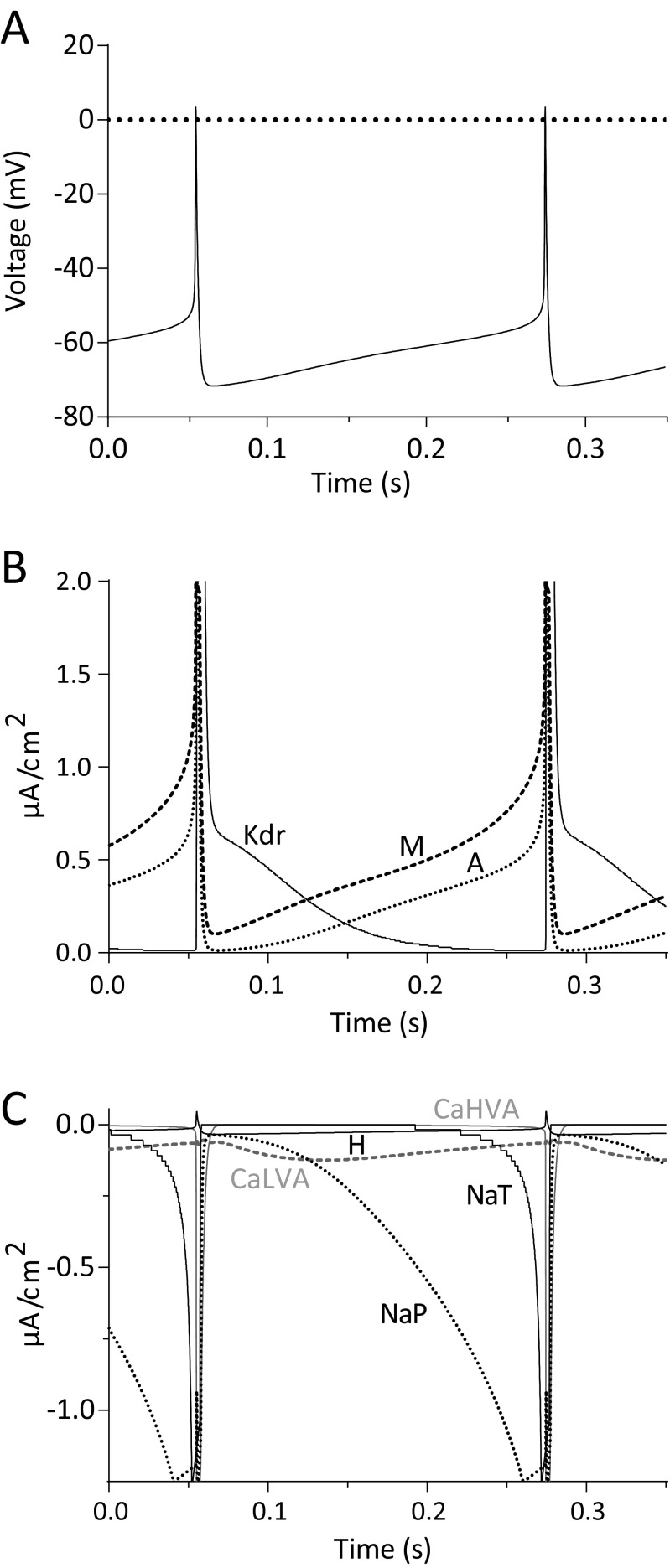
Contribution of individual ionic currents to interspike interval (ISI) repolarisation in model DA neuron. **a**: Simulation trace depicting the voltage trajectory during ISI between two successive APs in model DA neuron. **b**: Simulation trace depicting the current flowing through three individual outward potassium currents (A, M and K_dr_) during the ISI of two successive APs (voltage response as shown in A). Note that, the deactivation of the IK_dr_ facilitates repolarisation from AHP peak to AP threshold while the activation of the I_M_ and I_A_ potassium currents oppose repolarisation. **c**: Representative simulation traces depicting the kinetics of inward sodium, calcium and H-type currents during the ISI of two successive APs (voltage response as shown in A). Note that, a persistent sodium current (I_NaP_) activated shortly after the AHP peak giving rise to a strong inward current. Similarly, but displaying very different kinetics in activation and magnitude of response, I_CaLVA_ gave rise to a small but non-inactivating inward current during ISI. In contrast, I_CaHVA_ current did not activate at all during ISI, while the small magnitude activation of the I_H_ current (a declining inward conductance) gave rise to a net outward current during ISI.

**Fig. 10:**
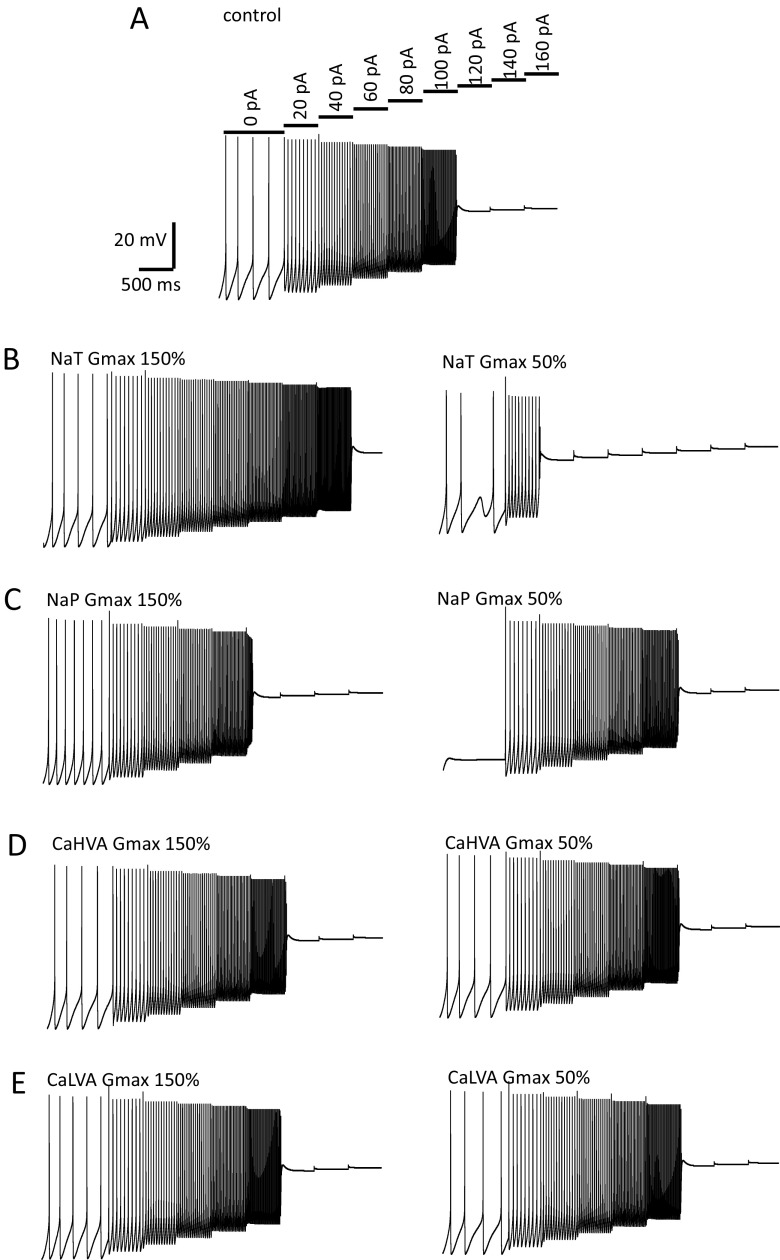
Contribution of individual ionic currents to depolarization block (DB) in model DA neuron. **a**. Representative 5 s simulation traces depicting the responses of model DA neurons to a sequence of depolarizing current injections (500 ms, black lines) leading to DB under basal control conditions. **b**. Strong effects of increasing (left, 150% of G_max_) and decreasing (right, 50% of G_max_) of I_NaT_ on the threshold of DB. **c**. Strong effects of increasing (left, 150% of G_max_) and decreasing (right, 50% of G_max_) of I_NaP_ on the threshold of DB. The effects of manipulating I_NaP_ on DB threshold were in opposite polarity from those observed by manipulation of I_NaT_ (see B above). **d**. No apparent effects of increasing (left, 150% of G_max_) or decreasing (right, 50% of G_max_) of I_CaHVA_ on the threshold of DB. **e**. No apparent effects of increasing (left, 150% of G_max_) or decreasing (right, 50% of G_max_) of I_CaLVA_ on the threshold of DB

Based on our experimental data, we constructed a single compartment, simplified, Hodgkin and Huxley- type DA neuron to compare model and *in vitro* neurophysiological behavior and explore the origin of autorhythmicity and pacemaking *in silico*. A summary of the mean values used for constructing the model DA neuron are given in Table 1. General equations used for the model DA neuron are given in the appendix. The model DA neuron exhibited autorhythmicity and fired APs at a pacemaker frequency of 4.6 Hz (Fig. 7a). AP characteristics (Fig. 7b) were measured and were compared with the corresponding mean values measured in the *in vitro* DA neuron in brain slices (Table 2, Fig. 7b). We found that the model DA neuron had similar AP characteristics and internal ratios to the native DA neurons (see also Dougalis et al. 2012) but notably had a faster action potential in width (at base) than *in vitro* vlPAG/DRN DA neurons. Model DA neuron exhibited an input resistance of 1.3 GΩ when subjected to small (−10 pA, 1000 ms) hyperpolarizing current injection in range with *in vitro* data (Dougalis et al. 2012). We injected a series of hyperpolarizing and depolarizing currents to the model DA neuron to validate its electrophysiological behavior (Fig. 7c, d) and to compare and contrast it with that of the *in vitro* DA neuron (Fig. 7e-h). Hyperpolarizing current injections were used to monitor the hyperpolarization-activated, cation current (I_H_ current-mediated voltage-sag) and I_A_ current-mediated delayed repolarization (quantified as the delay to firing a spike after the end of a hyperpolarizing current injection, Fig. 7c) which are the hallmarks of DA neuron electrophysiological behavior in vlPAG/DRN. Both responses were qualitatively and quantitatively similar to these obtained from *in vitro* brain slice preparation (Fig. 7g, h). Notably, the responses to the voltage-sag amplitude appeared fairly linear to those *in vitro* but the responses of the delayed depolarization produced an abruptly graded response at low current injections with no further increases detectable in delay to spiking thereafter. Upon injection of depolarizing pulses (Fig. 7d), the model DA neuron exhibited responses with similar gain (slope) in firing output to *in vitro* DA neurons only when given small magnitude current injection (10–40 pA, Fig. 7e-f). However, upon increasing the magnitude of the current injection (60–120 pA), the model DA neuron exhibited higher gain in output and fired at higher frequencies than that of the *in vitro* DA neurons while, it exhibited depolarization block (DB) at current magnitude within a similar range with *in vitro* data (Fig. 7e, f). Such behavior could be improved dramatically when needed by using a range of values reflecting the mean ± one standard deviation of the experimentally derived parameter during optimization without changing qualitatively the behavior and the origins of pacemaker (see later Figs. 8- 10). These data indicate that despite its apparent reductionist approach in the modelling of ion channel properties imposed herein, the model DA neuron captured and replicated well essential elements of vlPAG/DRN DA neuronal behavior as seen *in vitro*. Using this basal state of the model without further adjustments we then proceeded into further experiments attempting to understand the contribution of individual ion currents to firing frequency, autorhythmicity and threshold for depolarization block (Figs. 8- 10).

**Table 2:** A comparison of AP characteristics between *in vitro* and *in silico* DA neuron

**Parameter**	***in vitro*** **DA neuron**	**model DA neuron**
Firing frequency, (Hz)	4.1	4.6
AP threshold, (mV)	-43	-46
AP width at base, (ms)	4.2	2.5
AHP amplitude, (mV)	25	26
AHP maximum, (mV)	-65	-72
AHP repolarization, (mV/ms)	0.12	0.08

Comparative summary of characteristics for AP and AHP parameters in computer simulated model DA neuron and native DA neurons recorded in *in vitro* brain slices (values given as mean, AP characteristics were measured at AP threshold (width at base) or relative to the AP threshold (AP amplitude, AHP amplitude), while AHP repolarization was measured in the first 100 ms following the peak of the AHP. AP threshold was determined as the point where the slope of voltage change exceeded 10 mV/ms. All values have been corrected for liquid junction potentials

#### Contribution of ionic conductances in pacemaker firing frequency and expression of autorhythmicity

To evaluate the contribution of each ionic current to pacemaker firing frequency and neuronal autorhythmicity we used the model DA neuron to generate predictions by reducing the maximal conductance levels (G_max_ from 100% to zero) of each one individual conductance in turn (Fig. 8). We found that complete elimination of the I_A_ current caused a two-fold increase of model DA neuron basal pacemaker frequency (Fig. 8b). In contrast, eliminating the I_H_ current lead to a very small change (decrease) in firing frequency (Fig. 8c). Eliminating I_CaLVA_ and I_CaHVA_ calcium conductances decreased the model’s pacemaking frequency to different levels (Fig. 8d, e). The former, but not the latter, appeared as a strong regulator of firing frequency (causing an approximate 25% reduction in basal firing frequency) but complete and concomitant elimination of both calcium conductances did not stop spontaneous firing suggesting that calcium currents are not indispensable for vlPAG/DRN neuron’s spontaneous firing (Fig. 8f). Complete elimination of I_M_ current did not affect autorhythmicity either but instead it caused a two-fold increase in firing frequency (Fig. 8g). In contrast, small reductions of I_Kdr_ initially increased firing frequency (data not shown) but when the G_max_ value was reduced below 25% of its basal level, a stable non-oscillating depolarized potential above AP threshold (−35 mV) was attained accompanied by complete elimination of spontaneous firing even upon depolarizing current injections (Fig. 8h). Under these circumstances, even a small increase in the magnitude of the sustained outward I_M_ current (to 125% of the basal values, see online resource 3, also Wladyka and Kunze 2006; Shah et al. 2011) could rescue firing even when I_Kdr_ was set to zero (Fig. 8i) suggesting that although the I_kdr_ current modulates strongly autorhythmicity, its effect could be rescued by the small increase of another sustained potassium conductance in line with what would be expected from a Hodgkin and Huxley formalism, where the operation of sustained potassium conductances is a prerequisite for spontaneous firing. Elimination of I_NaP_ obliterated spontaneous firing but APs could still be observed under injection of depolarizing current (Fig. 8j). Eliminating the I_NaT_ current alone halted spontaneous firing (and depolarizing current injection- induced firing) but caused the appearance of a 6 mV oscillating background (at a frequency of 5.1 Hz) below AP threshold. Interestingly, concomitant elimination of both transient and persistent sodium currents stopped firing and produced a stable resting membrane potential nearly 10–20 mV more negative from the AP threshold (−55 to-64 mV, depending on the simulation conditions, Fig. 8k). The origin of the background oscillating potential seen in the absence of a I_NaT_ current was explored by inducing small reductions (to 75% of the value of G_max_) in either the I_NaP_ current (Fig. 8m) or the I_M_ current (Fig. 8n). Both treatments completely obliterated the background oscillation observed in the absence of the I_NaT_ current suggesting an interaction of the I_M_ and I_NaP_ persistent and opposing currents could give rise to a background oscillation under certain values/conditions in the subthreshold range when the I_NaT_ current is completely eliminated. These simulation data suggest that sodium, but not calcium, currents are primarily the mediators of autorhythmicity in the model DA neuron, while any individual potassium current alone (I_A_,I_Kdr_,I_M_), although strongly modulated pacemaker firing frequency, did not endow DA neurons with autorhythmicity. In contrast, it is a prerequisite that all individual sustained potassium currents are blocked simultaneously or reduced to less than 10% of their basal G_max_ value that causes spontaneous firing to cease due to the attainment of a depolarized steady state (*e.g.* Figure 8h).

#### Contribution of ionic conductances in repolarization during ISI

To understand in more detail the interplay of ionic currents in pacemaking and their contribution to the repolarization during the ISI we examined the trajectories of the each ionic current during this period in the model DA neuron (Fig. 9). We found that I_kdr_ potassium currents deactivated with an outward relaxation during ISI facilitating repolarization while the I_A_ and I_M_ currents activated during ISI thus opposing repolarization (Fig. 9b). Interestingly, the I_M_ current did not fully inactivate exhibiting a persistent outward current even at the peak of the AHP. We detected a rather small contribution (compared to I_Kdr_ and I_A_ type currents) from a inward current relaxation from the I_H_ current giving rise to a net outward current during ISI repolarisation. Importantly, I_CaHVA_ current did not contribute with any inward current during the ISI repolarization but I_CaLVA_ current gave rise to a small inward persistent current even at the peak of the AHP with growing contribution during the early phase of the ISI repolarisation. I_NaP_ remained inwardly active at the peak of the AHP and activated therafter strongly and quickly compared to I_CaLVA_. It displayed similar magnitude to the I_CaLVA_ current after 50–75 ms post AHP peak but I_NaP_ grew rapidly in inward contribution in the latter part of the ISI repolarisation, unlike the I_CaLVA_ that maintained a steady (not growing) non-inactivating inward contribution during late ISI repolarization. Collectively, these results highlight the importance of the persistent sodium current activation in driving autorhythmicity in vlPAG/DRN DA neurons and the opposing and facilitating roles of other inward and outward currents in setting the trajectory of voltage during ISI.

#### Contribution of individual ionic conductances in depolarization block (DB)

DA neurons of the vlPAG/DRN, unlike serotonin neurons in the vicinity, exhibit depolarization block (DB), a gradual adaptation and ultimately cessation of firing following challenge with an increasing magnitude depolarizing current pulse (Dougalis et al. 2012). Attainment of a DB state in DA neurons has been postulated to be important in the mode of action of antipsychotics in schizophrenia (Grace et al. 1997). Our model DA neuron also exhibited DB upon depolarizing current injections at a similar range of depolarizing current injections as native vlPAG/DRN DA neurons (see Fig. 7d, also Fig. 10a). We thus used the model DA neuron to understand how individual ionic conductances affect the magnitude of the current required (threshold) to achieved DB by focusing first on sodium and calcium currents. Reducing the G_max_ value (to 50%) of the I_NaT_ resulted in a reduction in the magnitude of the depolarized current required to produce DB (Fig. 10b) while increasing the G_max_ (to 150%) lead to exactly the opposite effect. In contrast, decreasing G_max_ value of the I_NaP_ (to 50%) resulted in no or a small increase in the DB threshold (Fig. 10c) while increasing G_max_ (1.5 times the G_max_) produced a lower threshold for DB. Similar reductions or increases in either I_CaHVA_ or I_CaLVA_ conductances did not affect the threshold for DB to the extent that sodium conductance modulation revealed in the model DA neuron (Fig. 10d-e) Similarly, moderate changes (reduction/increase) in the G_max_ of the I_H_ current as imposed previously for the sodium and calcium currents did not produce any significant effects on the threshold of depolarization block (online resource 5A). Changes in the G_max_ value for the I_A_ potassium current did not have an apparent effect on the DB threshold either (online resource 5B) although further changes beyond 50% modulated the threshold for DB in a manner similar to the effect of I_NaT_ (data not shown). Changes in the G_max_ values of the I_M_ current caused an effect similar but at much lower magnitude to the I_Kdr_ current (online resource 5C and D). The effects of small changes in the G_max_ value of the I_Kdr_ were more pronounced than the other potassium conductances where 50% increase or decrease in the G_max_ value strongly increased and decreased the DB threshold respectively (online resource 5D). The data suggest that the maximal somatic conductance of transient and persistent sodium currents distinctly modulates, in an opposing manner, the threshold for DB. Similarly, potassium currents reduce the threshold for DB to different degrees but calcium currents do not, indicating the operating complexity between sodium and potassium currents in setting the DB threshold for vlPAG/DRN DA neurons. Imposing larger reductions/increases than 50% of the maximum conductance of each individual current (*e.g.* reductions set to less than 20%) lead to identical changes to those reported above, but also revealed some modulating effects of the I_A_ and calcium currents (not seen before at 50 to 150% level changes imposed), suggesting that under more extreme challenges to the test conditions (longer steps, larger reductions/increases in the maximal conductance) other ionic currents may have a role in setting the DB threshold (data not shown).

## Discussion

DA neurons of the vlPAG and DRN fire action potentials at a frequency of 1–10 Hz but exhibit a much higher coefficient of variation in their firing patterns than SNc DA neurons (Dougalis et al. 2012). Their firing rate and pattern were found to be most similar to those of the VTA DA neurons (Dougalis et al. 2012). However, details of their intrinsic mechanism of operation including, the characteristics of ionic currents and their contribution to spontaneous firing are not known although such mechanisms have been long described for other midbrain DA neuronal groups (see Grace and Onn 1989; Silva et al. 1990; Puopolo et al. 2007; Khaliq and Bean 2010). To address this gap in knowledge we used voltage-clamp electrophysiology and pharmacology to isolate and describe a number of ionic currents operant on adult vlPAG/DRN DA neurons. We then analysed the data using a Hodgkin and Huxley formalism (Hodgkin and Huxley 1952) to construct a conductance-based computer model of a vlPAG/DRN DA neuron. We used this model to perform simulations and examine the involvement of individual ionic currents in DA neuron’s excitability by exploring their contribution in spontaneous firing, pacemaker frequency and threshold for spike frequency adaptation.

DA neurons of the midbrain express a hyperpolarization-activated cation current (I_H_ current) that has been long thought to be the hallmark of DA neuron electrophysiology. Recent evidence contest the absolute correlation of an I_H_ current with the DA phenotype as some non-DAergic neurons in the VTA also express I_H_ currents (see Margolis et al. 2006) while a subset of VTA DA neurons projecting to the prefrontal cortex (mesocortical projection pathway) do not express an I_H_ current (Lammel et al. 2008). In I_H_-expressing midbrain DA neurons, blockade of I_H_ conductance does not halt autorhythmicity, but instead leads to a reduction in pacemaker firing frequency only in a subtype of SNc, but not in VTA, DA neurons (Neuhoff et al. 2002; Puopolo et al. 2007; Khaliq and Bean 2010). DA vlPAG/DRN neurons expressed an I_H_ current of similar magnitude and kinetics with that of VTA, but not of that of the SNc, DA neurons (online resource 1). Blockade of I_H_ current in DA vlPAG/DRN neurons resulted, as expected, in ablation of the hyperpolarization-induced, slowly activating, inward current (under voltage-clamp) or the depolarizing voltage-sag (under current-clamp) but did not significantly affect basal firing frequency or pacemaker variability *in vitro*. The DA model and *in vitro* brain slice data were in good agreement regarding the magnitude and kinetics of the I_H_ current-mediated voltage-sag. In our DA model, reduction or complete removal of the I_H_ current did not halt autorhythmicity and did not have large effects on firing of the model DA neuron, although it resulted in a reduction in basal firing frequency (about 10%). This is largely because the V_50_ for activation of the I_H_ current (−115 mV) is more negative than the subthreshold potential that the neuron usually attains *in vitro* (normally up to −70 mV) and thus the I_H_ current does not get efficiently activated during AHP following an AP (estimated availability at −70 mV of less than 5%). The more positive V_50_ for I_H_ current activation of the SNc DA neurons (25 mV more positive than vlPAG/DRN or VTA DA neurons, see online resource 1) could potentially explain the effects of I_H_ current blockade on SNc DA neuron’s firing rate and the lack of effects in VTA DA neurons firing rate reported previously (Neuhoff et al. 2002; Puopolo et al. 2007¸ Khaliq and Bean 2010) or the lack of effects seen here for vlPAG/DRN DA neurons. Examination of the trajectory of activation of I_H_ current in the model DA neuron during the ISI suggests that a small I_H_ inward current declines steadily from the peak of AHP to AP threshold giving rise to a net outward current during ISI repolarization. The I_H_ current did not modulate the threshold for DB (online resource 5) further arguing that this current plays no significant role in vlPAG/DRN DA neuron excitability. These data taken together suggest that, similar to VTA DA neurons (Khaliq and Bean 2010), autorhythmicity of adult vlPAG/DRN DA neurons is largely independent of the I_H_ current which contributes little in setting their basal firing frequency or influencing neuronal excitability.

Repolarization of DA neurons during the ISI and tuning of pacemaker frequency in individual SNc and VTA neurons is dependent on expression of an A-type transient potassium current (I_A_ current, Silva et al. 1990; Liss et al. 2001; Koyama and Appel 2006a). The diversity of I_A_ current behavior is dependent on the different number of potassium transcripts that can contribute to the assembly of these channels. Kv4.1, Kv4.2, Kv4.3, Kv3.4 and Kv1.4 subunits have been reputed to give rise to transient I_A_-like currents but only the Kv4.3 transcripts were found expressed in SNc DA neurons (Liss et al. 2001). SNc DA neuron’s I_A_ currents are mediated by the Kv4.3 (long) transcript (heteropoda toxin 3-sensitive) where it seems to form functional I_A_ channels in combination with accessory protein KChip3 (but not KChip 1,2 or 4, see Liss et al. 2001). Using a two protocol subtraction method (as reported before, Koyama and Appel 2006a), we recorded outward transient currents sensitive to 4-AP blockade which possessed a voltage-dependent activation time constant consistent with previous reports (Liss et al. 2001; Koyama and Appel 2006a). However, in our study the activation V_50_ for the I_A_ current was found to be 30 and 20 mV more negative to what has been reported previously for SNc and VTA DA neurons respectively (Liss et al. 2001; Koyama and Appel 2006a), while the inactivation V_50_ was similar to the study of VTA DA neurons (approximately −90 mV, Koyama and Appel 2006a) and about 30 mV more negative to what has been reported in SNc DA neurons recorded under whole-cell conditions (Liss et al. 2001). Additionally, inactivation of the I_A_ currents in vlPAG/DRN DA neurons studied here was described better with the sum of two exponentials at potentials more positive than −30 mV, a fast (about 100 ms, voltage-dependent) and a slow (about 550 ms, voltage-independent), which differs kinetically from the reported monoexponential and voltage-independent fast decay (around 30–50 ms) of I_A_ currents in both SNc and VTA neurons *in vitro* (Silva et al. 1990; Liss et al. 2001; Koyama and Appel 2006a). The differences in the activation /inactivation range and kinetics of the inactivation of I_A_ currents described here with previous reports of I_A_ currents in VTA and SNc DA neurons can be attributed to differences in the preparations (*e.g.* physiological *versus* room temperature, brain slices *versus* dissociated neurons, whole-cell recordings *versus* outside-out patches) or inherently differential calcium influx kinetics (that may affect the properties of I_A_ currents recorded, see Koyama and Appel 2006a)and residual recruited currents. By exclusion of the above possibilities, the discrepancies can also represent a real difference in the gating properties of I_A_ currents in vlPAG/DRN DA neurons given that SNc and VTA DA neurons exhibit projection-specific differential physiological characteristics and greater diversity in their properties than previously envisaged (e.g see results from Lammel et al. 2008, 2011). The methodological differences between our study and the available studies (Liss et al. 2001; Koyama and Appel 2006a) could explain some of the differences observed in I_A_ current gating in vlPAG/DRN DA neurons. For example, I_A_ channels may be distributed both in somatic and dendritic domains but the latter are severed in dissociated neurons while often a mixture of somatic/extrasomatic channels is sampled in outside-out patch recordings leading to potential difference in the kinetics recorded (as acknowledged by Liss and colleagues, a 15 mV difference in inactivation V_50_ value was seen between whole-cell and outside-out patch recordings in SNc DA neurons). Also, in both the studies of Liss and colleagues and Koyama and Appel, only a single exponential was fitted and reported for I_A_ currents despite the fact that the currents could have had more complex inactivation properties (referred to as dominant fast time constant for SNc DA neurons in Liss et al. 2001; see also clear evidence of biexponential decay in I_A_ current recordings from dissociated VTA DA neurons in Koyama and Appel 2006a, figure 1A2). In our hands, VTA and vlPAG/DRN DA neurons exhibited similar I_A_ current behavior with slow (100–150 ms inactivation time constant), initially monoexponential decay (at more negative potentials than −40 mV) with biexponential inactivation apparent at more positive potentials than −30 mV on long steps (see tail currents in online resource 1). The available data derived from the interpretation of the three main studies of I_A_ current kinetics (Silva et al. 1990; Liss et al. 2001 and Koyama and Appel 2006a) and the evidence from projection specific studies of DAergic populations (see Lammel et al. 2008, 2011) suggest that I_A_ current properties in SNc and VTA DA neurons are not similar but a systematic study of the I_A_ current properties of projection-specific DA groups is still lacking making further interpretation and comparison to our data difficult. Interestingly, in our hands, 4-AP sensitive currents also exhibited a bi-exponential inactivation (online resource 2) in a similar manner to those recorded with the two protocol subtraction method. In our experiments, 4-AP increased a background conductance leading to typical current ‘crossing’ upon current subtraction in good agreement to what has been reported before by others in DA neurons (see Liss et al. 2001; Khaliq and Bean 2008) indicating some of the potential inherent difficulties in using 4-AP as a tool to kinetically describe I_A_ currents. Despite this, a third method of isolating the I_A_ conductance (recordings in the presence of TEA as in Silva et al. 1990) also showed that the I_A_ currents in vlPAG/DRN DA neurons exhibit a strong biexponential decay with the fast exponential decay being twice as long (approximately 70 ms, albeit voltage-independent) compared to the reports of Liss and colleagues or Koyama and Appel suggesting that the biexponential properties seen here should most likely reflect I_A_ current physiological operation in adult vlPAG/DRN DA neurons in brain slices. Also, all three methods of I_A_ current isolation used here resulted in commensurate V_50_ values for the activation of the current (around −55 to −60 mV) suggesting further that the kinetics of activation described here are likely to reflect I_A_ current gating *in situ* in brain slices rather than an artefact of the isolation method. The contribution of other background residual currents that could potentially contaminate our I_A_ recording (*e.g.* a calcium-activated potassium conductance, I_H_ current) cannot be excluded, although such a proposal could not directly explain the biexponential decay of the 4-AP-sensitive currents seen here or the biexponential slow decay seen in TEA recordings. Using our voltage-clamp data, we modelled a reduced version of I_A_ current with a single, voltage-dependent exponential decay according to the two protocol subtraction method. Our model DA neuron exhibited well-defined delayed repolarization upon hyperpolarizing current steps to > −80 mV under current-clamp that was carried forward by the I_A_ conductance and was quantitatively similar to delayed repolarization to firing recorded in brain slices *in vitro*. The model DA neuron predicted that a reduction in I_A_ currents will lead to an increase in pacemaker firing frequency an effect seen previously in SNc DA neurons using blockade with heteropoda toxin-3 (Liss et al. 2001). I_A_ currents develop a strong outward current during ISI repolarization from the peak of the AHP to AP threshold. Although abolition of the I_A_ current lead to a two-fold increase in firing frequency, it did not stop spontaneous firing, suggesting that I_A_ current is not essential for autorhythmicity. Interestingly, blocking the I_A_ current in the DA neuron model did not strongly reduce the threshold for DB (online resource 5) suggesting that I_A_ currents are major determinants of neuronal excitability in DA vlPAG/DRN neurons but do not exhibit high propensity in modulating BD threshold at least when exposed to the sequences and range of depolarizing pulses studied herein. Indeed, longer pulses (> 1000 ms) or greater changes in G_max_ rendered the DA model neuron more susceptible to the effects of the I_A_ current (data not shown) suggesting that under certain, more robust, conditions DB threshold is also modulated by I_A_ currents. In a similar manner to I_A_ potassium currents, blockade of I_Kdr_ positively modulated firing frequency in the DA neuron model but also modulated strongly the threshold for DB (online resource 5). The deactivation of I_Kdr_ following the AHP had a dramatic effect in membrane repolarization during ISI repolarization to AP threshold. We also show here that outward potassium currents recorded in vlPAG/DRN DA neurons are a composite of different non-inactivating/slowly-inactivating currents including a non-inactivating potassium conductance activating at more negative potentials than typical I_Kdr_ currents and mediated through XE991-sensitive KCNQ (Kv7) channels suggesting that an M-current (I_M_) is also expressed in DA vlPAG/DRN neurons with similar properties to that expressed in VTA DA neurons (online resource 3, see also Koyama and Appel 2006b, see also Hansen et al. 2006; Drion et al. 2010). The complete abolition of I_Kdr_ current renders the neuron highly depolarized and stops autorhythmicity. This is largely because I_Kdr_ current is the only persistent outward current in this neuron that can help facilitate effective repolarization after an AP by virtue of its deactivation. Despite the three-fold lower maximal conductance of the I_M_ current compared to the I_Kdr_ current used in our model, increasing the I_M_ current contribution by just minute amounts (10–25%) rescues the spontaneous firing of the DA neuron in the complete absence of an I_Kdr_ current, leading to the establishment of low frequency rhythmic APs and plateaus in the DA model neuron. The model suggests that I_M_ current does not inactivate completely following AHP, thus providing a steady background outward current even at the peak of the AHP. This explains the fact that increase of I_M_ current rescues neuron’s spontaneous firing in the absence of I_Kdr_ while abolition of the I_M_ current dramatically increased firing frequency (and induced a depolarising current *in vitro*) but did not halt spontaneous firing of the DA model neuron (as partially seen in Koyama and Appel 2006b also see opposite effects of I_M_ current opener retigabine in Hansen et al. 2006). The I_M_ current also had an effect on the threshold for DB in a manner similar, but far less stronger to the I_Kdr_ current (see online resource 5). Our model suggests that the I_M_ current is efficiently activated during ISI repolarization and in tandem with the I_A_ current provides a strong outward current delaying the repolarization to AP threshold. These results collectively suggest that potassium currents strongly modulate neuronal excitability of vlPAG/DRN neurons but are not necessary individually *per se* for the expression of autorhythmicity. It is rather that, the complete negation of sustained outward currents like I_Kdr_ and I_M_ simultaneously, is the prerequisite to render the neuron inactive and unable to generate rhythmically APs in line with a Hodgkin and Huxley formalism (Hodgkin and Huxley 1952).

Calcium currents play an important role in the pacemaking process in a number of diverse neuronal preparations, from midbrain SNc DA neurons to hypothalamic GABAergic suprachiasmatic nucleus neurons (*e.g.* Jackson et al. 2004; Khaliq and Bean 2010). Unlike SNc DA neurons, VTA DA neurons express a sodium ‘persistent’ background current that dominates over the rather small calcium current in the subthreshold range during ISI (Puopolo et al. 2007; Khaliq and Bean 2010). In our study, similar to the results obtained by Khaliq and Bean (2010) in VTA DA neurons, blockade of voltage-gated calcium currents by low calcium/high magnesium solutions lead to an increase in the pacemaker frequency in vlPAG/DRN DA neurons. Although such a treatment could have also reduced calcium-dependent potassium conductances leading to an increase in firing rate (but see also studies of Wolfart and Roeper 2002; Deignan et al. 2012), it demonstrates that pacemaking can persist in the absence of calcium indicating that calcium is not critical in driving spontaneous firing of vlPAG/DRN DA neurons. Interestingly, removal of calcium from the extracellular solution lead to small, albeit statistically insignificant reduction in the pacemaker variability unlike what would be expected from blockade of calcium-dependent potassium currents (see Wolfart et al. 2001), implying that this change represents potentially the algebraic sum of negating all calcium-driven and calcium-dependent currents (with potentially opposing roles in pacemaker variability, see Poetschke et al. 2015) in vlPAG/DRN DA neurons. Our experiments indicate that both transient LVA and slowly-inactivating HVA (through a calcium release-mediated calcium inactivation) calcium currents are expressed in vlPAG/DRN DA neurons with properties similar to those described previously in other systems (Brevi et al. 2001; Jackson et al. 2004) but also in DA neurons (Kang and Kitai 1993a, 1993b). The slowly inactivating HVA component was sensitive to nimodipine, indicating that an L-type calcium current is partly responsible for the inward conductance recorded (online resource 4). The LVA component had characteristics reminiscent of a T-type current (see Kang and Kitai 1993a), was resistant to nimodipine (online resource 4) but sensitive to cadmium. Our model suggests that full blockade of the I_CaLVA_ currents, did not halt DA model neuron autorhythmicity or affect its threshold for DB but reduced basal pacemaker firing frequency by nearly 30%. I_CaLVA_ currents contributed with a small (compared to potassium currents) but constant inward current (that did not fully inactivate during or shortly after AP) during ISI repolarization to AP threshold. In contrast, to the I_CaLVA_ currents, I_CaHVA_ currents failed to contribute with any inward current during ISI repolarization to AP threshold, remaining essentially inactive during ISI and activating only during the AP. Blocking I_CaHVA_ current did not halt autorhythmicity of DA model neuron (alone or in combination with blockade of the I_CaLVA_ currents) and did not affect pacemaker firing frequency or contribute to any degree in setting the threshold for DB (even on longer steps as described for the I_A_ current). This is perhaps expected by I_CaHVA_ current that has on average nearly 30 mV more positive activation midpoint in comparison to the I_CaLVA_ current, explaining why the latter can affect neuronal operation in the subthreshold range. Our data collectively suggest that calcium currents are not required for spontaneous firing on the model DA neuron but I_CaLVA_ currents may have a role in modulating neuronal excitability in the opposite direction and less strongly than potassium conductances.

Sodium currents play an important role in the pacemaking activity of VTA DA neurons driving repolarization during ISI through a voltage-dependent, TTX-sensitive and a voltage-independent, TTX-resistant component (see Khaliq and Bean 2010). They are less important in the pacemaking of SNc DA neurons where calcium currents dominate and drive repolarization during the ISI (Puopolo et al. 2007). Blocking TTX-sensitive sodium channels in the brain slice lead to a stable, non-oscillating, resting membrane potential in vlPAG/DRN DA neurons, in a similar manner to what has been reported previously for VTA DA neurons (Khaliq and Bean 2010) and in contrast to the reported behavior of SNc DA neurons (see Nedergaard et al. 1993; Mercuri et al. 1994; Chan et al. 2007; Puopolo et al. 2007; Guzman et al. 2009). In the model DA neuron, blockade of both fast and persistent sodium conductances lead to the establishment of a hyperpolarized stable non-oscillating membrane potential in the same manner as in the *in vitro* brain slice. I_NaT_ and I_NaP_ currents start activating at different time points and rates following AHP during ISI repolarization and are adominant force driving DA neurons towards AP threshold. I_NaP_ current does not fully inactivate following an AP and rapidly activate during ISI repolarization relatively fast when compared to I_NaT_ current that is engaged significantly only in the last 50 ms before the occurrence of a AP under normal conditions. Blockade of I_NaP_ current alone halted spontaneous firing but depolarizing current injections restored the firing capacity of the DA model neuron suggesting that the expression of spontaneous firing is affected by the I_NaP_ current but not the capacity to generate APs in response to external inputs. Blockade of I_NaT_ currents alone stopped all spiking behavior on model DA neuron, inclusive of depolarization-induced spiking, and lead to a relatively hyperpolarized (compared to AP threshold) membrane potential with evidence of background oscillations (at 5 Hz) that were dependent on an interaction of the I_NaP_ current and the I_M_ current. Indeed, small reductions (*i.e.* 10–25%) in either I_M_ current or I_NaP_ current during the absence of a I_NaT_ current obliterated the oscillatory background behavior and resulted in a steady-state non-oscillating hyperpolarizing potential without affecting the overall working of the model. Such a behavior was not seen in the brain slice preparation suggesting that it could be related to the direct interaction of the activation kinetics of outward and inward persistent conductances in the model but its physiological significance (if any) is not currently known. Perhaps, studies utilizing dynamic voltage-clamp techniques to selectively negate I_NaP_ or I_NaT_ conductance in conjunction with pharmacology could help enormously in clarifying the distinct roles of transient and persistent sodium currents (or any interactions of the latter with the I_M_ seen in the model DA neuron) in setting the properties of *in vitro* adult vlPAG/DRN DA neurons. Finally, using the DA model neuron we found that both transient and persistent sodium currents affected strongly DB threshold albeit exhibiting opposing effects. In good agreement with our study, Tucker et al. (2012) also found that reduction in somatic transient sodium currents reduces the DB threshold in a both *in vitro* and model SNc DA neuron (Tucker et al. 2012). During our voltage-clamp analysis, we could not reliably detect any discernable sodium current that persisted in the presence of TTX and thus we infer that TTX-resistant currents are not operant on vlPAG/DRN DA neurons, although it is also possible that we may have missed this small component. Our data collectively suggest that sodium currents endow DA vlPAG/DRN neurons with autorhythmicity by slowly activating during ISI repolarization and effectively bringing the neuron towards the AP threshold for firing.

In conclusion, the data presented here extend our previous physiological characterization (Dougalis et al. 2012) and argue that DA neurons of the vlPAG/DRN express autorhythmicity in the absence of synaptic transmission *via* the interplay of potassium and sodium currents without the absolute need of calcium currents. The properties of the ionic currents recorded here (I_H_ current, I_A_ current), the lack of small oscillating potentials in the presence of sodium channel blockers taken together with the mechanisms for autorhythmicity (reliance more on sodium rather than calcium currents) also support further the idea that vlPAG/DRN DA neurons are operationally similar to VTA, rather than SNc, DA neurons. In particular, the properties of a slowly inactivating I_A_ current in conjunction with the small and slowly activating I_H_ current described herein pinpoint that vlPAG/DRN DA neurons are most similar to prefrontal cortex or medial shell of nucleus accumbens projecting DA neurons (see Lammel et al. 2008, 2011). Given the importance of vlPAG/DRN DA neurons in arousal, reward and nociception, it would be interesting in the future to understand how the electrical properties and the synaptic activity arriving at these neurons may be modulated by sleep-wake cycles, drugs of abuse (e.g cocaine, heroin), or how it may change in models of nociception (*e.g.* following spinal cord injury or in neuropathic pain).

### Electronic supplementary material

Online resource 1 Comparative properties of I_H_ current activation in SNc, VTA and vlPAG/DRN DA neurons. **a**. Representative electrophysiological traces recorded in voltage-clamp mode (holding voltage −62 mV, 10 mV incremental hyperpolarizing steps to −132 mV) depicting the activation of the I_H_ current in SNc, VTA and vlPAG/DRN DA neurons. **b**. Comparison of the voltage-dependence of the activation of the I_H_ current (% I/I_max_) for SNc, VTA and vlPAG/DRN DA neurons. Note the more positive mean activation V_50_ for SNc neurons (−92 mV, *n* = 7) as opposed to VTA and vlPAG/DRN neurons (VTA,-121 mV; vlPAG/DRN,-121 mV, *n* = 7 and 6 respectively). **c**. Comparison of the voltage-dependence of the activation time constant of the I_H_ current for SNc, VTA and vlPAG/DRN DA neurons. Note the similarity of the mean activation time constant (at −132 mV) of VTA and vlPAG/DRN neurons (VTA, 397 ms; vlPAG/DRN, 260 ms, *n* = 7 and 6 respectively) as opposed to the much faster kinetics of I_H_ current activation in SNc neurons (114 ms, *n* = 7). (JPEG 39 kb)

High Resolution Image (EPS 1420 kb)

Online resource 2 Comparative properties of I_A_ currents isolated *via* three different methods in vlPAG/DRN DA neurons. **a**. A second, slow, inactivation time constant (τ_slow_) could be fitted to the two protocol subtracted I_A_ currents recorded at more positive potentials than −30 mV. Unlike the fast inactivation constant (τ_fast_) that exhibited voltage sensitivity and faster kinetics at more positive holding potentials (Fig. 2h), τ_slow_ was largely voltage-independent and accounted for about half of the current’s amplitude (A_max_) at a potential of +8 mV (mean τ_fast_, 51 ms; mean τ_slow_ 562 ms at +8 mV, *n* = 6). **b**. Constructed steady-state activation curve (G/G_max_ against holding voltage) after digital subtraction of the 4-AP (2 mM) sensitive current (as shown in Fig. 2b, c). The 4-AP sensitive conductance saturated well within the test voltage range and exhibited a mean V_50_ and slope of −61.4 ± 0.9 mV and 7.1 ± 0.8 mV respectively (*n* = 3). The residual conductance (after 4-AP subtraction) did not saturate well within our voltage range and exhibited a much more depolarized V_50_ estimated 30 to 50 mV more positive than the 4-AP sensitive conductance (data not shown). **c**. Voltage dependence of the inactivation time constant (τ_fast_ and τ_slow_) for the 4-AP sensitive currents. Note that both inactivation time constants were voltage sensitive, becoming faster at more positive voltages. The second inactivation constant (τ_slow_) was consistently evident at holding voltages more positive than −30 mV. Mean τ_fast_ and τ_slow_ was 63 ± 5 ms and 335 ± 34 ms at +8 mV (*n* = 3), values similar to the results obtained with the two protocol subtraction method (see Fig. 2i and online resource 2A). **d**. Contribution of inactivation time constants (τ_fast_ and τ_slow_) to current amplitude expressed as a percentage of maximum current amplitude (A_max_) at different holding voltages. Note that 4-AP sensitive currents exhibited a slow inactivation time constant (τ_slow_) activating more positively than −30 mV and contributing to a mean of 27 ± 7% and 49 ± 5% of A_max_ at −22 mV and +8 mV respectively (*n* = 3) similar to the results obtained with the two protocol subtraction method (see above online resource 2A). **e**. Constructed steady-state activation curve (G/G_max_ against holding voltage) for the I_A_ currents isolated *via* single activation protocol (protocol 1 as shown in Fig. 2d) in the presence of 10 mM TEA (traces as shown in Fig. 2j). The I_A_ currents saturated within the test voltage range and exhibited a mean V_50_ and slope of −55.6 ± 1.0 mV and 15.1 ± 1.1 mV respectively (*n* = 5). **f**. Voltage dependence of the inactivation time constant (τ_fast_ and τ_slow_) for the I_A_ current isolated *via* a single activation protocol (protocol 1 as shown in Fig. 2d) in the presence of 10 mM TEA (as shown in Fig. 2j). Note that using this method, I_A_ currents exhibited consistently bi-exponential inactivation with time constants that were largely voltage insensitive (compare −72 mV and +8 mV, *n* = 5, *P* > 0.05, paired t-test). The second inactivation constant (τ_slow_) was evident at holding voltages more positive than −60 mV. Mean τ_fast_ and τ_slow_ values were 56 ± 12 ms and 309 ± 47 ms respectively at +8 mV (*n* = 5) similar to the results obtained with the two protocol subtraction method and the 4-AP-sensitive currents isolated (see Fig. 2i and online resource 2A, C). **g**. Contribution of inactivation time constants (τ_fast_ and τ_slow_) to current amplitude expressed as a percentage of maximum current amplitude (A_max_) at different holding voltages. Note that I_A_ currents isolated *via* a single activation protocol (protocol 1 as shown in Fig. 2d) in the presence of 10 mM TEA (as shown in Fig. 2j) exhibited a slow inactivation time constant (τ_slow_) activating more positively than −60 mV and contributing to a mean of 35 ± 3% and 59 ± 5% of A_max_ at −22 mV and +8 mV respectively (*n* = 5) analogous to the results obtained with the two protocol subtraction method and the 4-AP-sensitive currents isolated (see Fig. 2d, i and online resource 2A, D). (JPEG 54 kb)

High Resolution Image (EPS 1588 kb)

Online resource 3 Properties of an I_M_ potassium current in vlPAG/DRN DA neurons. **a**. Typical averaged electrophysiological traces during a standard deactivation protocol for the M-type potassium current (I_M_) (Koyama and Appel 2006b). Neurons were recorded with a KGlu based-internal solution in the presence of TTX (1 μM). Neurons were held at −72 mV and were given a depolarizing prepulse to −32 mV for 1 s to fully activate the M current before stepping down from −42 to −72 mV for 1 s to record the resultant I_M_ current deactivation tail. Currents were sensitive to the KCNQ blocker XE991 (30 μM) and to TEA (10 mM, *n* = 4 data not shown) suggesting the involvement of KV7.2 subunits in mediating the responses (scale bars, 50 pA, 250 ms). **b**. Overlay of electrophysiological traces showing details of I_M_ current relaxation measurement (step hyperpolarization from −32 to −62 mV) before and after the application of XE991 (30 μM) (scale bars, 10 pA, 250 ms). **c**. Voltage-dependence of I_M_ tail current amplitude and deactivation time constant. Deactivation time constant was significantly slower at more positive potentials (mean of 62 ± 6 ms at −62 mV and 235 ± 17 ms at −42 mV, *n* = 13, *P* < 0.0001, paired t-test) in close agreement with reports of I_M_ currents in VTA DA neurons (Koyama and Appel 2006b). **d**. Percentage inhibition caused by XE991 (30 μM) on the amplitude of the I_M_ deactivation tail current measured at different holding voltages following a prepulse to −32 mV (black squares). For comparison, the amplitude of the sustained outward current recruited after a depolarizing step from −72 to −32 mV is given (single point white square). Note that XE991 not only reduced the I_M_ current relaxation but also the sustained outward current upon depolarizing pulses (mean 67%, *n* = 4). (JPEG 36 kb)

High Resolution Image (EPS 767 kb)

Online resource 4 The effects of nimodipine on I_BaHVA_ and I_BALVA_ currents in vlPAG/DRN DA neurons. **a**. Fast voltage-ramp (200 mV/s, −107 to +13 mV) depicting barium currents in the presence and absence of the L-type calcium channel blocker nimodipine (10 μM). Nimodipine-sensitive barium current was fitted with a single Boltzmann function (bottom to top) and had a steady state activation V_50_ of −22.1 mV and a slope of 4.2. **b**. Percentage block of barium LVA and HVA currents by nimodipine at different holding voltages studied by a series of voltage steps (from a holding potential of −87 mV, 10 mV increments from −77 to −7 mV). Nimodipine (10 μM) inhibited the persistent HVA barium current by 47 ± 8% (at −37 mV) but left the transient LVA barium current relatively unaffected (4 ± 3% reduction at −57 mV, *n* = 3). (JPEG 32 kb)

High Resolution Image (EPS 1460 kb)

Online resource 5 Contribution of I_H_, I_A_, I_M_ and I_kdr_ on DB threshold in vlPAG/DRN DA neurons. Representative 5 s simulation traces depicting the responses of model DA neurons to a sequence of depolarizing current injections (500 ms, as shown in Fig. 10a) leading to DB. **a**. Lack of effect of I_H_ on the threshold of DB. **b**. Lack of major effects of I_A_ on the threshold of DB. **c**. Modulation of DB threshold by I_M._ **d**. Strong modulation of DB threshold by I_kdr_ (JPEG 56 kb)

High Resolution Image (EPS 2337 kb)

## Appendix

### General model features

General equations for the construction and the workings of the NEURON simulator and the implementation of neuronal model within NEURON have been described extensively previously (see Hines and Carnevale 1997) and are briefly discussed and presented below together with specific equations used for the working of vlPAG/DRN DA neuron.

To follow the time evolution of neuronal membrane potential (V_m_) for a neuron with a membrane capacitance C_m_ one must obtain the sum of each operant ionic current (denoted as (*ion*)) flowing through ionic channels on the neuron’s membrane. The general eq. (6) (current balance equation) below describes the relationship of membrane voltage and ionic current flowing through individual ionic channels for any neuron with membrane capacitance C_m_.

6 $$ Cm\frac{dVm}{dt}=-\sum I(ion) $$

The ionic current (I_(ion)_) passing through any ionic channel is both a voltage and time-dependent function. It can be calculated as the product of the *ion*’s maximal conductance (G_max_) times the driving force at any given potential (V_m_-E_rev_, where E_rev_ is the reversal potential for the conductance, Eq. (7))

7 $$ I(ion)= G \max {\left( Vm, t\right)}^{\ast}\left( Vm- Erev\right) $$

The ionic currents in the model were described by standard, Hodgkin-Huxley–type equations in which generalized Boltzmann-type equations defined the voltage- and time-dependent activation and inactivation of conductances. Specifically, ionic conductances were evaluated by solving general eq. (8). Conductance (G_(ion)_) of any given *ion* at any given time t at any given V_m_ can be described as the product of the ion’s maximal conductance (G_max_) times the activation function (A_(ion)_) times the inactivation function (B_(ion)_) of the *ion.* A_(ion)_ (V_m_,t) and B_(ion)_ (V_m_,t) are functions describing the voltage and time dependence of activation and inactivation associated with G_(ion)_, while p and n is the power to which A_(ion)_ and B_(ion)_were raised and was taken as the standard value for a given ionic channel. *P* and n can be physically represented by the number of gating stages/steps for the ion channel activation and inactivation function respectively and were given a value of 3 and 1 for sodium channels and 4 and zero for potassium channel activation and inactivation functions respectively as described previously by Hodgkin and Huxley (1952).

8 $$ G(ion)= G \max {(ion)}^{\ast }{A}^p(ion){\left( Vm, t\right)}^{\ast }{B}^n(ion)\left( Vm, t\right) $$

A_(ion)_and B_(ion)_were given by the solution to the general differential eqs. (9) and (10) below:

9 $$ \frac{dA(ion)}{dt}=\frac{A\infty (ion)- A(ion)}{\tau A(ion)} $$

10 $$ \frac{dB(ion)}{dt}=\frac{B\infty (ion)- B(ion)}{\tau B(ion)} $$

where A^**00**^_(ion)_ and B^**00**^_(ion)_ are the voltage-dependent, steady-state values of the activation and inactivation functions, respectively; and τ_A(ion)_ and τ_B(ion)_ are the voltage-dependent time constants of the activation and inactivation functions, respectively. The values of A^**00**^_(ion)_ and B^**00**^_(ion)_ were determined from the general eqs. (11) and (12) below:

11 $$ A\infty (ion)=\frac{1}{1+ \exp \left(\frac{Vm- hA}{sA}\right)} $$

12 $$ B\infty (ion)=\frac{1}{1+ \exp \left(\frac{Vm- hB}{sB}\right)} $$

where V_m_ is the membrane voltage, whereas h_A_, h_B)_ and s_A_, s_B)_ determine the midpoints (V_50_) and slopes of the steady-state activation and inactivation functions, respectively. The voltage dependence of the activation and inactivation time constants (τ_A(ion)_ and τ_B(ion)_) were represented by one of the two following functions respectively. Otherwise, τ was held constant.

13 $$ \tau \mathrm{A}(ion)=\frac{\tau \mathrm{A} \max -\tau \mathrm{A} \min }{1+ \exp \left(\frac{Vm- h\tau A}{s\tau A}\right)}+\tau \mathrm{A} \min $$

14 $$ \tau B(ion)=\frac{\tau B \max -\tau B \min }{1+ \exp \left(\frac{Vm- h\tau B}{s\tau B}\right)}+\tau B \min $$

where V_m_ is the membrane voltage; τ_A max_, τ_A min_ and τ_B max_, τ_B min_ are the maximal and minimal values for the activation and inactivation time constants, respectively; hτ_A,_ hτ_B_ and sτ_A_, sτ_B_ determine the (V_50_) midpoints and slopes of the time constant functions, respectively. Equation (13) & (14) were used since the voltage dependence of the time constants was best fit by a sigmoid in the physiological range.
