# Supplementary figures and images for: Ionic currents influencing spontaneous firing and pacemaker frequency in dopamine neurons of the ventrolateral periaqueductal gray and dorsal raphe nucleus (vlPAG/DRN): A voltage-clamp and computational modelling study
Source: J Comput Neurosci. 2017 Apr 3;42(3):275–305. doi: 10.1007/s10827-017-0641-0 (PMC5403876; doi:10.1007/s10827-017-0641-0)

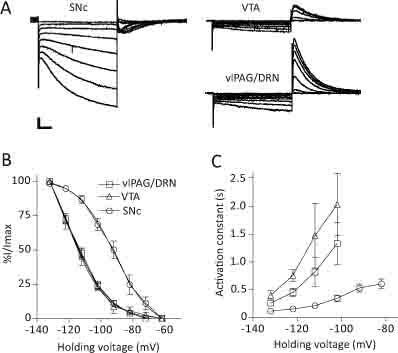

Supplement: Supplementary file 1 — Comparative properties of IH current activation in SNc, VTA and vlPAG/DRN DA neurons. a. Representative electrophysiological traces recorded in voltage-clamp mode (holding voltage −62 mV, 10 mV incremental hyperpolarizing steps to −132 mV) depicting the activation of the IH current in SNc, VTA and vlPAG/DRN DA neurons. b. Comparison of the voltage-dependence of the activation of the IH current (% I/Imax) for SNc, VTA and vlPAG/DRN DA neurons. Note the more positive mean activation V50 for SNc neurons (−92 mV, n = 7) as opposed to VTA and vlPAG/DRN neurons (VTA,-121 mV; vlPAG/DRN,-121 mV, n = 7 and 6 respectively). c. Comparison of the voltage-dependence of the activation time constant of the IH current for SNc, VTA and vlPAG/DRN DA neurons. Note the similarity of the mean activation time constant (at −132 mV) of VTA and vlPAG/DRN neurons (VTA, 397 ms; vlPAG/DRN, 260 ms, n = 7 and 6 respectively) as opposed to the much faster kinetics of IH current activation in SNc neurons (114 ms, n = 7). (JPEG 39 kb) [file 10827_2017_641_Fig11_ESM.jpg]

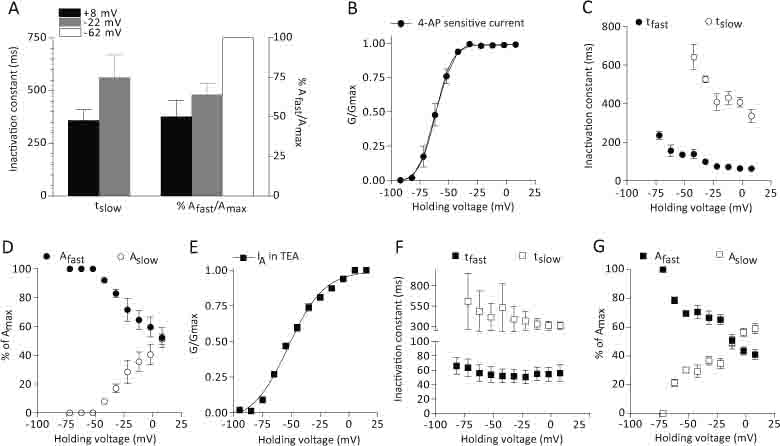

Supplement: Supplementary file 3 — Comparative properties of IA currents isolated via three different methods in vlPAG/DRN DA neurons. a. A second, slow, inactivation time constant (τslow) could be fitted to the two protocol subtracted IA currents recorded at more positive potentials than −30 mV. Unlike the fast inactivation constant (τfast) that exhibited voltage sensitivity and faster kinetics at more positive holding potentials (Fig. 2h), τslow was largely voltage-independent and accounted for about half of the current’s amplitude (Amax) at a potential of +8 mV (mean τfast, 51 ms; mean τslow 562 ms at +8 mV, n = 6). b. Constructed steady-state activation curve (G/Gmax against holding voltage) after digital subtraction of the 4-AP (2 mM) sensitive current (as shown in Fig. 2b, c). The 4-AP sensitive conductance saturated well within the test voltage range and exhibited a mean V50 and slope of −61.4 ± 0.9 mV and 7.1 ± 0.8 mV respectively (n = 3). The residual conductance (after 4-AP subtraction) did not saturate well within our voltage range and exhibited a much more depolarized V50 estimated 30 to 50 mV more positive than the 4-AP sensitive conductance (data not shown). c. Voltage dependence of the inactivation time constant (τfast and τslow) for the 4-AP sensitive currents. Note that both inactivation time constants were voltage sensitive, becoming faster at more positive voltages. The second inactivation constant (τslow) was consistently evident at holding voltages more positive than −30 mV. Mean τfast and τslow was 63 ± 5 ms and 335 ± 34 ms at +8 mV (n = 3), values similar to the results obtained with the two protocol subtraction method (see Fig. 2i and online resource 2A). d. Contribution of inactivation time constants (τfast and τslow) to current amplitude expressed as a percentage of maximum current amplitude (Amax) at different holding voltages. Note that 4-AP sensitive currents exhibited a slow inactivation time constant (τslow) activating more positively than −30 mV and contributing to a m [file 10827_2017_641_Fig12_ESM.jpg]

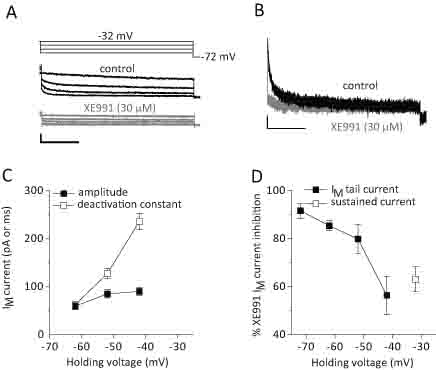

Supplement: Supplementary file 5 — Properties of an IM potassium current in vlPAG/DRN DA neurons. a. Typical averaged electrophysiological traces during a standard deactivation protocol for the M-type potassium current (IM) (Koyama and Appel 2006b). Neurons were recorded with a KGlu based-internal solution in the presence of TTX (1 μM). Neurons were held at −72 mV and were given a depolarizing prepulse to −32 mV for 1 s to fully activate the M current before stepping down from −42 to −72 mV for 1 s to record the resultant IM current deactivation tail. Currents were sensitive to the KCNQ blocker XE991 (30 μM) and to TEA (10 mM, n = 4 data not shown) suggesting the involvement of KV7.2 subunits in mediating the responses (scale bars, 50 pA, 250 ms). b. Overlay of electrophysiological traces showing details of IM current relaxation measurement (step hyperpolarization from −32 to −62 mV) before and after the application of XE991 (30 μM) (scale bars, 10 pA, 250 ms). c. Voltage-dependence of IM tail current amplitude and deactivation time constant. Deactivation time constant was significantly slower at more positive potentials (mean of 62 ± 6 ms at −62 mV and 235 ± 17 ms at −42 mV, n = 13, P < 0.0001, paired t-test) in close agreement with reports of IM currents in VTA DA neurons (Koyama and Appel 2006b). d. Percentage inhibition caused by XE991 (30 μM) on the amplitude of the IM deactivation tail current measured at different holding voltages following a prepulse to −32 mV (black squares). For comparison, the amplitude of the sustained outward current recruited after a depolarizing step from −72 to −32 mV is given (single point white square). Note that XE991 not only reduced the IM current relaxation but also the sustained outward current upon depolarizing pulses (mean 67%, n = 4). (JPEG 36 kb) [file 10827_2017_641_Fig13_ESM.jpg]

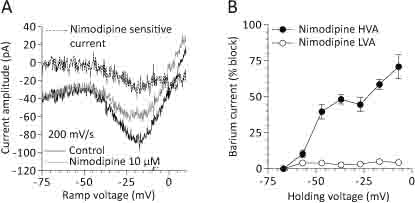

Supplement: Supplementary file 7 — The effects of nimodipine on IBaHVA and IBALVA currents in vlPAG/DRN DA neurons. a. Fast voltage-ramp (200 mV/s, −107 to +13 mV) depicting barium currents in the presence and absence of the L-type calcium channel blocker nimodipine (10 μM). Nimodipine-sensitive barium current was fitted with a single Boltzmann function (bottom to top) and had a steady state activation V50 of −22.1 mV and a slope of 4.2. b. Percentage block of barium LVA and HVA currents by nimodipine at different holding voltages studied by a series of voltage steps (from a holding potential of −87 mV, 10 mV increments from −77 to −7 mV). Nimodipine (10 μM) inhibited the persistent HVA barium current by 47 ± 8% (at −37 mV) but left the transient LVA barium current relatively unaffected (4 ± 3% reduction at −57 mV, n = 3). (JPEG 32 kb) [file 10827_2017_641_Fig14_ESM.jpg]

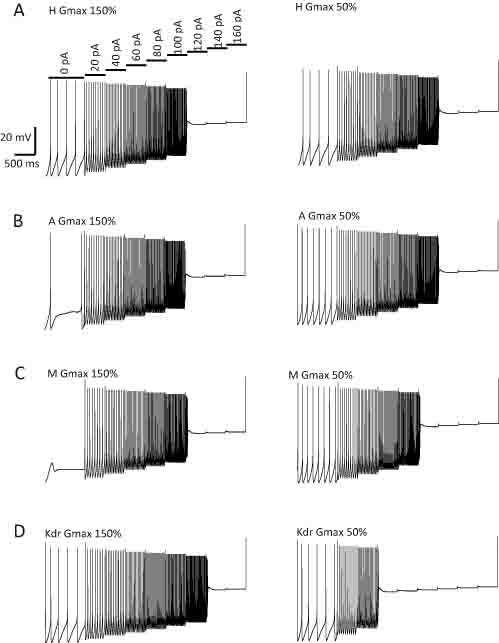

Supplement: Supplementary file 9 — Contribution of IH, IA, IM and Ikdr on DB threshold in vlPAG/DRN DA neurons. Representative 5 s simulation traces depicting the responses of model DA neurons to a sequence of depolarizing current injections (500 ms, as shown in Fig. 10a) leading to DB. a. Lack of effect of IH on the threshold of DB. b. Lack of major effects of IA on the threshold of DB. c. Modulation of DB threshold by IM. d. Strong modulation of DB threshold by Ikdr (JPEG 56 kb) [file 10827_2017_641_Fig15_ESM.jpg]
